# LNA-anti-miR-150 alleviates renal interstitial fibrosis by reducing pro-inflammatory M1/M2 macrophage polarization

**DOI:** 10.3389/fimmu.2022.913007

**Published:** 2022-08-05

**Authors:** Xiangnan Hao, Junjun Luan, Congcong Jiao, Cong Ma, Zixuan Feng, Lingzi Zhu, Yixiao Zhang, Jingqi Fu, Enyin Lai, Beiru Zhang, Yanqiu Wang, Jeffrey B. Kopp, Jingbo Pi, Hua Zhou

**Affiliations:** ^1^ Department of Nephrology, Shengjing Hospital of China Medical University, Shenyang, China; ^2^ Department of Urology, Shengjing Hospital of China Medical University, Shenyang, China; ^3^ Program of Environmental Toxicology, School of Public Health, China Medical University, Shenyang, China; ^4^ Department of Physiology, School of Basic Medical Sciences, Zhejiang University School of Medicine, Hangzhou, China; ^5^ Kidney Disease Section, NIDDK/NIH, Bethesda, MD, United States

**Keywords:** LNA-anti-miR-150, folic acid, SOCS1/JAK1/STAT1, M1/M2 macrophage polarization, renal fibrosis

## Abstract

Renal interstitial fibrosis (RIF) is a common pathological feature contributing to chronic injury and maladaptive repair following acute kidney injury. Currently, there is no effective therapy for RIF. We have reported that locked nuclear acid (LNA)-anti-miR-150 antagonizes pro-fibrotic pathways in human renal tubular cells by regulating the suppressor of cytokine signal 1 (SOCS1)/Janus kinase (JAK)/signal transducer and activator of transcription (STAT) pathway. In the present study, we aimed to clarify whether LNA-anti-miR-150 attenuates folic acid-induced RIF mice by regulating this pathway and by reducing pro-inflammatory M1/M2 macrophage polarization. We found that renal miR-150 was upregulated in folic acid-induced RIF mice at day 30 after injection. LNA-anti-miR-150 alleviated the degree of RIF, as shown by periodic acid–Schiff and Masson staining and by the expression of pro-fibrotic proteins, including alpha-smooth muscle actin and fibronectin. In RIF mice, SOCS1 was downregulated, and p-JAK1 and p-STAT1 were upregulated. LNA-anti-miR-150 reversed the changes in renal SOCS1, p-JAK1, and p-STAT1 expression. In addition, renal infiltration of total macrophages, pro-inflammatory M1 and M2 macrophages as well as their secreted cytokines were increased in RIF mice compared to control mice. Importantly, in folic acid-induced RIF mice, LNA-anti-miR-150 attenuated the renal infiltration of total macrophages and pro-inflammatory subsets, including M1 macrophages expressing CD11c and M2 macrophages expressing CD206. We conclude that the anti-renal fibrotic role of LNA-anti-miR-150 in folic acid-induced RIF mice may be mediated by reducing pro-inflammatory M1 and M2 macrophage polarization *via* the SOCS1/JAK1/STAT1 pathway.

## Introduction

Renal interstitial fibrosis (RIF), a common pathological feature of end-stage kidney disease (ESKD), causes personal and economic burdens worldwide. As there is a lack of effective therapeutic agents to slow or halt the progression of RIF, the prevalence of ESKD remains unacceptably high (1, 2). Clarifying the mechanisms of RIF and discovering novel therapeutic targets are urgent needs.

Renal infiltration of macrophages is a key factor in the progression of acute kidney injury to chronic kidney disease (CKD) (3, 4). In the kidney, acute kidney injury activates molecular pathways that initially stimulate the differentiation of macrophages into the M1 phenotype. M1 macrophages contribute to the decline of renal function and development of renal fibrosis (5, 6). CD206+ M2 macrophages are also strongly associated with renal fibrosis in human and experimental kidney diseases (7).

MicroRNAs (miRs) also regulate gene expression during each stage of macrophage development, from myelopoiesis, through polarization and effector function. Furthermore, they regulate macrophage polarization signals and metabolic functions (8, 9). The role of miRNA regulation of macrophages in kidney disease has also been studied. For instance, miR-374b-5p contributes to renal inflammation and promotes M1 macrophage activation by directly targeting the suppressor of cytokine signal 1 (SOCS1) during renal ischemia/reperfusion injury progression (10). Furthermore, miR-30a-5p inhibition alleviates cardiac injury following viral myocarditis by shifting the macrophages toward a M2 phenotype *via* SOCS1 upregulation (11). We have reported that a miR-150 antagonist reversed SOCS1/Janus kinase (JAK)/signal transducer and activator of transcription (STAT) pathway in co-cultures of human kidney 2 (HK-2) cells and macrophages, and we further showed that LNA-anti-miR-150 alleviates folic acid-induced renal fibrosis in mice (12). However, the relationship among miR-150, macrophages, and renal fibrosis and the underlying mechanisms has not been characterized.

In the present study, we aimed to clarify the relationship among miR-150, pro-inflammatory M1, and M2 macrophage polarization and renal fibrosis. We further investigated the effects of LNA-anti-miR-150 on SOCS1/JAK/STAT and the infiltration of macrophages, including M1 and M2 subtypes, in folic acid-induced renal fibrosis mice. We show that LNA-anti-miR-150 reduces pro-inflammatory M1 and M2 polarization and that this is mediated by the SOCS1/JAK/STAT pathway.

## Materials and methods

### Animal experimental design

The animal studies were approved in advance by the Animal Care and Use Committee of China Medical University (15052111) and were performed following NIH Animal Care and Use Guidelines. Male ICR mice (12 weeks old, 35–40 g) were purchased from Beijing Vital River Laboratory Animal Technology Co. Ltd. (Beijing, China), housed at three mice per cage, and allowed free access to standard food and drinking water. The mice were maintained under a 12-h light/dark cycle with a fixed temperature of 23 ± 1°C and humidity (55–70%). The mice were injected intraperitoneally with 250 mg/kg folic acid (Sigma-Aldrich, MO, USA) in a vehicle of 0.3 mM NaHCO_3_ (0.2 ml/mouse) or the vehicle alone. After confirming acute kidney injury (AKI) based on significantly increased blood urea nitrogen (BUN) and serum creatinine, the mice (*n* = 24) were divided into four groups: (1) normal control (NC), (2) folic acid alone, (3) folic acid + scrambled LNA, and (4) folic acid + LNA-anti-miR-150. The mice were injected subcutaneously with LNA-anti-miR-150 or scrambled LNA (Exiqon, MA, USA), starting at day 2, at a dose of 2 mg/kg twice weekly, for a total of eight doses over 4 weeks.

### Sample collection

Peripheral blood samples were collected on days 0, 2, and 30 following folic acid administration. On day 30, the mice were anesthetized, blood samples were collected from the abdominal aorta, and kidneys were collected after perfusion with phosphate-buffered saline to remove intrarenal blood as previously described (12). Plasma was isolated from blood samples and was stored at -80°C. The kidneys were divided into four parts: 1/2 of the left kidney was fixed into 4% paraformaldehyde and the tissue was embedded in paraffin, 1/2 of the left kidney was put into optimal cutting temperature compound (Sakura, CA, USA) and stored at -80°C, and the right kidney was divided into one vertical and horizontal cut, and four pieces of kidney tissue, including intact renal cortex and medulla, were stored at -80°C for isolation of total protein and RNA.

### Serology chemistry

BUN and serum creatinine (Scr) were measured using commercial kits (Njjcbio, China) and an Architect c16000 clinical chemistry analyzer (Abbott, Chicago, IL, USA).

### Histology studies

Kidney sections (3 µm) were cut from mouse paraffin-embedded kidney tissue blocks and stained with hematoxylin and eosin (H&E), periodic acid–Schiff (PAS), and Masson (Solarbio, China) stains. On PAS-stained sections, tubular injury was scored with a semi-quantitative approach by an observer masked to the sample identities (13). For each mouse, we arbitrarily selected 100 tubules at ×400 magnification. Each tubular profile was assigned one of five categories according to the following criteria: 0, normal; 1, areas of tubular epithelial cell swelling, vacuolar degeneration, necrosis, and/or desquamation involving <25% of tubular profile; 2, similar changes involving ≥25 but <50% of tubular profile; 3, similar changes involving ≥50% but <75% tubular profile; and 4, similar changes involving ≥75% tubular profile. NIH Image J was used to semi-quantify the renal fibrosis area on Masson-stained sections as previously described (12).

### Immunocytochemistry staining

Antigen retrieval for immunohistochemistry (IHC) staining was performed on 3-μm mouse kidney sections, which were deparaffinized, rehydrated, and incubated in citrate buffer for 20 min at 95°C. Non-specific binding was blocked with 10% normal goat serum (Zsbio, Beijing, China) for 15 min at 37°C. The slides were incubated overnight at 4°C with antibodies against α-smooth muscle (α-SMA), fibronectin (FN), SOCS1, phospho-Janus kinase-1 (p-JAK1), phosphorylated-signal transducer and activator of transcription-1 (p-STAT1), CD11c, CD68, and CD206 (**Table 1**). This was followed by incubation with biotin-conjugated goat anti-rabbit immunoglobulin IgG (Zsbio, China) for 15 min at 37°C. The sections were exposed to streptavidin-conjugated peroxidase (Zsbio, China) for 15 min at 37°C. The reaction products were visualized using a diaminobenzidine kit (Zsbio, China). Images were captured by microscopy (Nikon Corporation, Tokyo, Japan). Eight IHC parameters were quantified by Image J software (NIH, Bethesda, MD, USA) as previously reported (14).

**Table 1:** Antibodies used in Western blotting and immunohistochemistry.

Protein	Company	Catalog	Host	Application	Dilution
a-SMA	Cell Signaling Technology	19245	Rabbit	WB	1:1,000
				IHC	1:500
Fibronectin	Abcam	ab2413	Rabbit	WB	1:1,000
				IHC	1:100
SOCS1	Cell Signaling Technology	3950s	Rabbit	WB	1:1,000
SOCS1	Bioss	bs0113R	Rabbit	IHC	1:100
JAK1	Cell Signaling Technology	3344	Rabbit	WB	1:1,000
p-JAK1	Cell Signaling Technology	74129	Rabbit	WB	1:1,000
p-JAK1	Affinity	AF2012	Rabbit	IHC	1:100
STAT1	Cell Signaling Technology	14994	Rabbit	WB	1:1,000
p-STAT1	Cell Signaling Technology	9167	Rabbit	WB	1:1,000
				IHC	1:800
CD68	Affinity	DF7518	Rabbit	WB	1:1,000
				IHC	1:200
CD11c	Cell Signaling Technology	97585	Rabbit	WB	1:1,000
				IHC	1:100
CD206	Abcam	ab64693	Rabbit	WB	1:1,000
CD206	Servicebio	GB11062	Rabbit	IHC	1:1,000
a-Tubulin	Cell Signaling Technology	2125	Rabbit	WB	1:1,000

a-SMA, a-smooth muscle actin; FN, fibronectin; SOCS1, suppressor of cytokine signaling 1; JAK1, janus kinase-1; p-JAK1, phosphor-janus kinase-1; STAT1, signal transducer and activators of transcription-1; p-STAT1, phosphor-signal transducer and activators of transcription-1; CD68, cluster of differentiation 68; CD11c, cluster of differentiation 11c; CD206, cluster of differentiation 206; WB, Western blotting; IHC, immunohistochemical staining.

### Western blotting

Kidney total proteins were extracted using radioimmunoprecipitation assay buffer with protease inhibitors, and protein concentrations were determined by bicinchonic acid assay (Beyotime, China). Equal amounts of total protein from kidney tissues (50 μg) were separated by SDS-PAGE and transferred onto polyvinylidene fluoride membranes (Millipore Immobilon-P, MA, USA). After blocking with 5% milk, the membranes were incubated at 4°C overnight with primary antibodies against a-SMA, FN, SOCS1, JAK-1, p-JAK1, STAT1, p-STAT1, CD68, CD11c, CD206, and α-tubulin (**Table 1**). After washing the blots, goat anti-rabbit immunoglobulin G (IgG) was added for 1 h at room temperature. Antibody–antigen binding was detected by High-sig ECL Western blotting Substrate (Wanlei, Shenyang, China) and visualized by the Tanon 5500 imaging system (Shanghai, China). Protein loading variation was normalized by α-tubulin. Blot density was analyzed by NIH Image J software (Bethesda, MD, USA). Protein level is expressed as the ratio of blot density from an individual protein to its housekeeping antibody.

### qPCR

Total RNAs were isolated from the frozen kidney tissues using TRIzol reagent (Life Technologies, Carlsbad, CA, USA) according to instructions, and RNA concentration was measured with Nanodrop 2000 (ThermoFisher, Waltham, MA, USA). RNA (50 ng) was subjected to reverse transcription using Prime Script RT Reagent Kit and followed by PCR with SYBR Premix Ex Taq (Takara, China) for the mRNA of proinflammatory cytokines, including CXCL1 and CXCL10 for M1 macrophages and CCL17 and CCL26 for M2 macrophages, as well as miR-150. Primers were designed using Primer Express (Applied Biosystems, CA, USA) and synthesized by Life Technologies (Shanghai, China). Real-time fluorescence signal was detected with QuantStudio 6 Flex quantitative real-time PCR system (Applied Biosystems). Beta-actin and small nucleolar miRNA (Sno202) were used as endogenous controls for mRNA and mouse miR-150, respectively (**Table 2**). Relative levels of mRNA and miR-150 were calculated using the 2^−ΔΔCt^ method (ΔCt: Ct value of endogenous control gene – Ct of individual target gene).

**Table 2:** Sequence of RNA and RNAi used in the present study.

Gene	Host	Forward (5′–3′)		Reverse (5′–3′)	Application
mmu-miR-150	Mouse	TCTCCCAACCCTTGTACCAGTG			qPCR
Sno202	Mouse	GCTGTACTGACTTGATGAAAGTACT			qPCR
CXCL1	Mouse	CTGGGATTCACCTCAAGAACATC	CAGGGTCAAGGCAAGCCTC		qPCR
CXCL10	Mouse	CCAAGTGCTGCCGTCATTTTC	GGCTCGCAGGGATGATTTCAA		qPCR
CCL17	Mouse	GACGACAGAAGGGTACGGC	GCATCTGAAGTGACCTCATGGTA		qPCR
CCL26	Mouse	TTCTTCGATTTGGGTCTCCTTG	GTGCAGCTCTTGTCGGTGAA		qPCR
Beta-actin	Mouse	TTCCTTCTTGGGTATGGAAT	GAGCAATGATCTTGATCTTC		qPCR
LNA-anti-miR-150	Mouse	TACAAGGGTTGGGAG			RNAi *in vivo*
Scrambled LNA	Mouse	TAGAAGGGTGGTGAC			RNAi *in vivo*

LNA, locked nucleic acid; RNAi, RNA interference; CXCL, C-X-C motif chemokine ligand; CCL, chemokine (C–C motif) ligand.

### Statistical analysis

Prism 9.0 (GraphPad, San Diego, CA, USA) software was used for statistical analysis and graphing. Quantitative data are expressed as mean ± SD. Difference between the two groups was analyzed by a *t*-test. A value of *p <*0.05 was considered as statistically significant.

## Results

### miR-150 increased in folic acid-induced RIF mice

**Figure 1:**
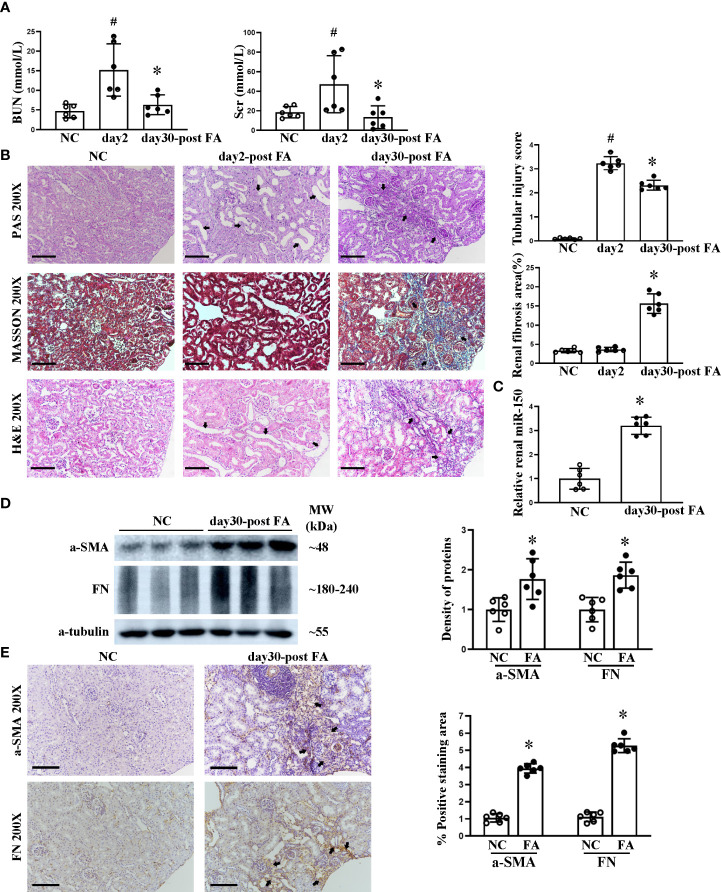
Course of folic acid-induced kidney injury and renal miR-150 expression in mice. The dynamic changes of renal function indicated by blood urea nitrogen and serum creatinine **(A)**. Histological morphological changes by periodic acid–Schiff, Masson, and H&E staining as well as semi-quantification of tubular injury score and renal fibrosis area **(B)** in mice after folic acid (FA) injection. Relative renal expression of miR-150 determined by qPCR **(C)**. Shown are the renal expression of profibrotic protein including a-SMA and fibronectin by Western blotting and the density of the bands **(D)** and immunohistochemical (IHC) staining and their respective semi-quantitative analysis at day 30 after FA administration **(E)**. Data are presented as mean ± SD, *n* = 6. In **(A, B)**, ^#^ *p* < 0.05, day 2 post-FA mice vs. normal control (NC) mice. In **(A)**, *p < 0.05, day 30 post-FA vs. day 2 post-FA. In **(B–E)**, *p < 0.05, day 30 post-FA vs. NC mice. For PAS, Masson, H&E, and IHC, magnification = ×200 and scale bar = 100 μm.

First, we confirmed that folic acid induced AKI, followed by renal fibrosis. At day 2 following folic acid injection, AKI was detected, with elevated levels of BUN and Scr. Until day 30, BUN and Scr returned close to baseline (**Figure 1A**). AKI was confirmed on histology, with renal tubular lumen expansion, tubular epithelial cell vacuolization and brush border loss, renal tubular epithelial cell detachment from basement membrane, and a sparse infiltrate of inflammatory cells seen on PAS, Masson, and H&E staining. At day 30, renal tubular atrophy, patchy fibrosis of medulla rays, and severe infiltration of inflammatory cells appeared. Tubular injury score and percent renal fibrosis area showed the severity of tubular injury and renal fibrosis (**Figure 1B**). Based on this disease course, we focused on day 30, when RIF was striking. miR-150 was upregulated in the kidney at day 30 after FA injection, quantitated by qPCR analysis (**Figure 1C**). We examined the expression of pro-fibrotic proteins in kidney tissues. The expression of α-SMA and fibronectin was increased in RIF mice compared to control mice by Western blotting and immunohistochemical staining; semi-quantification of expression is shown in **Figures 1D, E**.

### LNA-anti-miR-150 alleviated RIF

**Figure 2:**
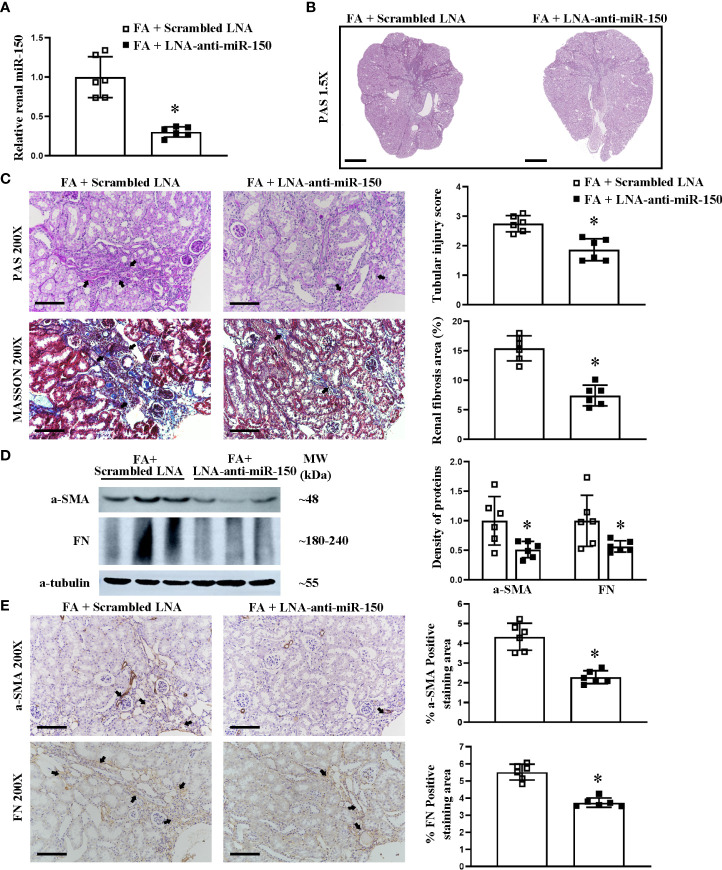
LNA-anti-miR-150 inhibited the renal miR-150 expression and attenuated the renal injured production of profibrotic proteins in renal interstitial fibrosis mice. Renal miR-150 levels were inhibited by LNA-anti-miR-150 **(A)**. Morphologic damage including kidney surface scarring **(B)** and patchy fibrosis along the medullary rays was improved, as assessed by PAS staining; semi-quantification. Renal fibrosis improvement was also seen on Masson staining; semi-quantification is shown **(C)**. Renal profibrotic proteins including a-SMA and fibronectin analyzed by Western blotting **(D)** and immunohistochemical staining **(E)**. For PAS staining, magnification ×1.5, scale bar = 1 mm **(B)**. For PAS, Masson, and immunohistochemical staining, magnification = ×200, scale bar = 100 μm. For all graphs, data are presented as mean ± SD, *n* = 6. *p < 0.05, FA + LNA-anti-miR-150 group vs. FA + scrambled LNA group.

We have previously reported that LNA-anti-miR-150 is delivered to the mouse kidneys following its systemic administration (12, 14, 15). Therefore, we investigated the efficacy of LNA-anti-miR-150 on folic acid-induced RIF at day 30 after eight doses of the injections. LNA-anti-miR-150 reduced the upregulation of renal miR-150 on day 30 (**Figure 2A**). Furthermore, PAS and Masson staining and semi-quantitative analysis of fibrosis area showed that LNA-anti-miR-150 attenuated renal fibrosis. Specifically, there was a reduction in the extent of regions of patchy fibrosis adjacent to the medullary rays as demonstrated at low magnification (**Figure 2B**). Moreover, the tubular injury score was reduced, and there were fewer infiltrating inflammatory cells on PAS-stained sections as well as reduced fibrosis area on Masson stain (**Figure 2C**). The increased levels of fibrotic proteins, including a-SMA and fibronectin, were reversed by LNA-anti-miR-150, compared to the scrambled LNA, as assessed by Western blotting and immunostaining (**Figures 2D, E**).

### LNA-anti-miR-150 ameliorated RIF through the SOCS1/JAK1/STAT1 pathway

**Figure 3:**
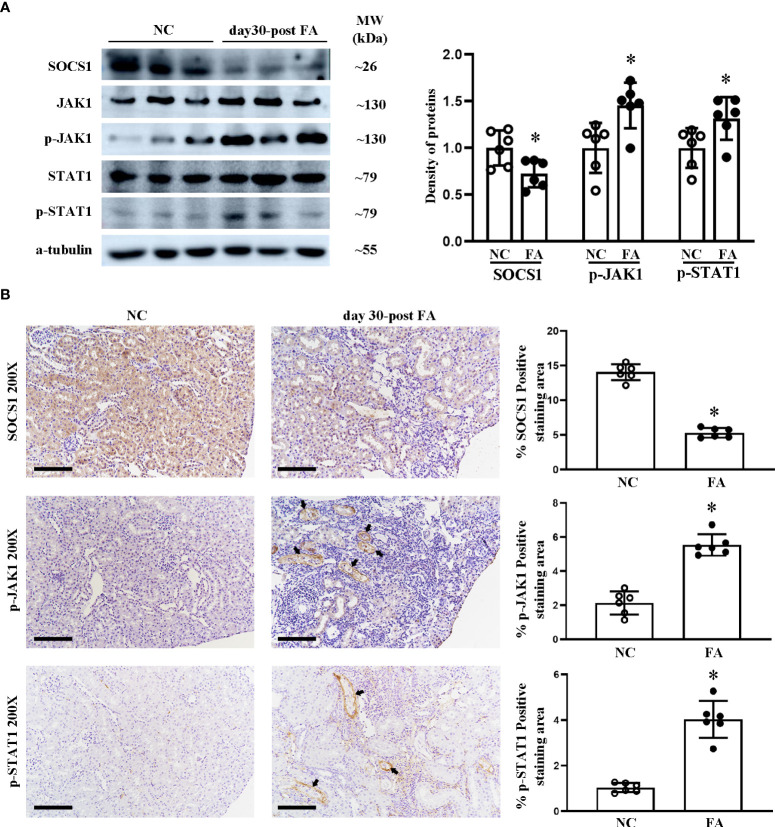
Renal expression of proteins on the SOCS1/JAK1/STAT1 pathway in folic acid (FA)-induced renal interstitial fibrosis mice. Western blotting **(A)** of SOCS1, JAK1, p-JAK1, STAT1, and p-STAT1 and the density of the blots. Immunohistochemical (IHC) staining **(B)** of SOCS1, p-JAK1, and p-STAT1 and the percentage of positive-staining area of each of the abovementioned proteins. For IHC, magnification = ×200, scale bar = 100 μm. For all graphs, data are presented as mean ± SD, *n* = 6. *p < 0.05, day 30 post-FA group vs. NC group.

**Figure 4:**
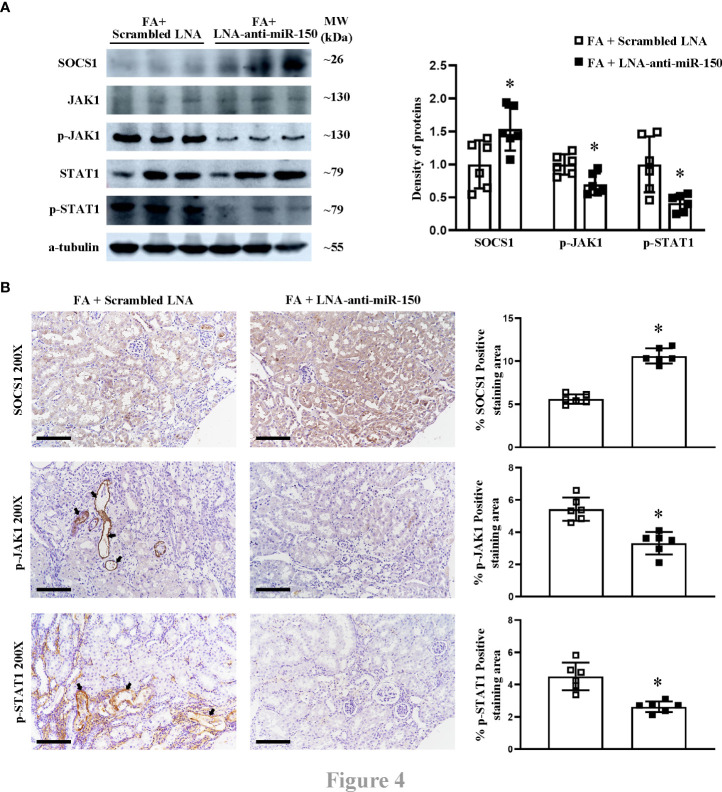
The effect of LNA-anti-miR-150 on the protein expression of SOCS1/JAK1/STAT1 pathway in renal interstitial fibrosis mice. Western blotting **(A)** demonstrated the renal protein levels of SOCS1, JAK1, p-JAK1, STAT1, and p-STAT1 and the density of the blots. Immunohistochemical (IHC) staining **(B)** of SOCS1, p-JAK1, and p-STAT1 and the percentage of positive-staining area of each of the abovementioned proteins are shown. For immunohistochemical staining, magnification = ×200, scale bar = 100 μm. For all graphs, data are presented as mean ± SD, *n* = 6. *p < 0.05, FA + LNA-anti-miR-150 group vs. FA + scrambled LNA group.

We previously reported in a study using the luciferase reporter gene that miR-150 mimic can downregulate SOCS1 mRNA, which encodes the suppressor of cytokine signaling 1 (16). We have previously reported that LNA-anti-miR-150 regulates the SOCS1/JAK1/STAT1 pathway in experiments using HK-2 cells co-cultured with macrophages (12). To verify whether this pathway also operates *in vivo*, we examined the renal protein levels of SOCS1, JAK1, p-JAK1, STAT1, and p-STAT1 in folic acid-induced RIF mice by Western blotting and immunohistochemical staining as well as their respective semi-quantification. SOCS1 was downregulated, and p-JAK1 and p-STAT1 were upregulated in the kidneys of folic acid-injected mice compared to those of normal mice (**Figures 3A, B**). LNA-anti-miR-150 intervention reverted these changes in the protein expression of the SOCS1/p-JAK1/p-STAT1 pathway (**Figures 4A, B**). Thus, the SOCS1/p-JAK1/p-STAT1 pathway contributes to renal fibrosis in folic acid-induced RIF mice.

### Renal infiltration of the polarized M1 and M2 macrophages was increased in RIF mice

**Figure 5:**
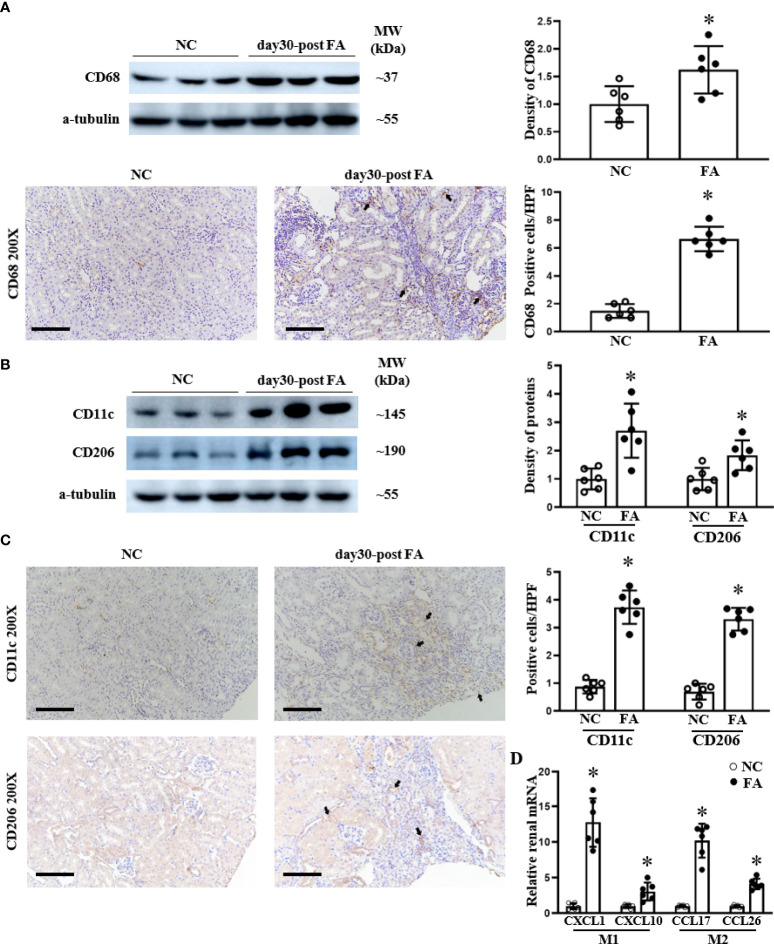
The infiltration of total macrophages, M1 macrophages, and M2 macrophages increased in the kidneys of renal interstitial fibrosis mice. Western blots and immunohistochemical (IHC) staining of the renal expression of CD68 protein, a typical biomarker for total macrophages **(A)**, are shown. M1 macrophage polarization is indicated by CD11c, and M2 macrophage polarization is indicated by CD206 expression as analyzed using Western blotting **(B)** and IHC staining and their respective semi-quantification **(C)**. The renal mRNA levels of M1-related cytokines CXCL1and CXCL10 as well as M2-related cytokines CCL17 and CCL26 were assessed by qPCR **(D)**. For IHC staining, magnification = ×200, scale bar = 100 μm. For all graphs, data are presented as mean ± SD, *n* = 6. *p < 0.05, day 30 post-FA group vs. NC group.

The JAK/STAT pathway contributes to the activation and polarization of macrophages (17). To clarify the renal infiltration of macrophages and the polarization of pro-inflammatory M1 and M2 polarization in folic acid-induced RIF mice, we assessed CD68+ (total) macrophages, CD11c+ M1 macrophages, and CD206+ M2 macrophages. CD68 macrophage protein was increased at day 30 in folic acid-injected RIF mice by both Western blotting and immunohistochemical staining (**Figure 5A**).

We investigated the polarization of macrophages to M1 and M2 states. Pro-inflammatory CD11c and CD206 proteins were upregulated in RIF mice compared to control mice as assessed by Western blotting (**Figure 5B**) and by immunohistochemical staining, which demonstrated macrophage localization to fibrotic areas (**Figure 5C**). In addition, we examined the expression of pro-inflammatory cytokine characteristics of M1 and M2 macrophages. The renal expression of CXCL1 and CXCL10 (secreted by M1 macrophages) was upregulated in RIF mice compared to normal mice, which was assessed at the mRNA level by qPCR (**Figure 5D**). Similarly, the renal mRNA levels of CCL17 and CCL26 cytokines secreted by M2 macrophages were also increased on qPCR in RIF mice compared to control mice (**Figure 5D**).

### LNA-anti-miR-150 inhibited the polarization of renal macrophage M1 and M2 in RIF mice

**Figure 6:**
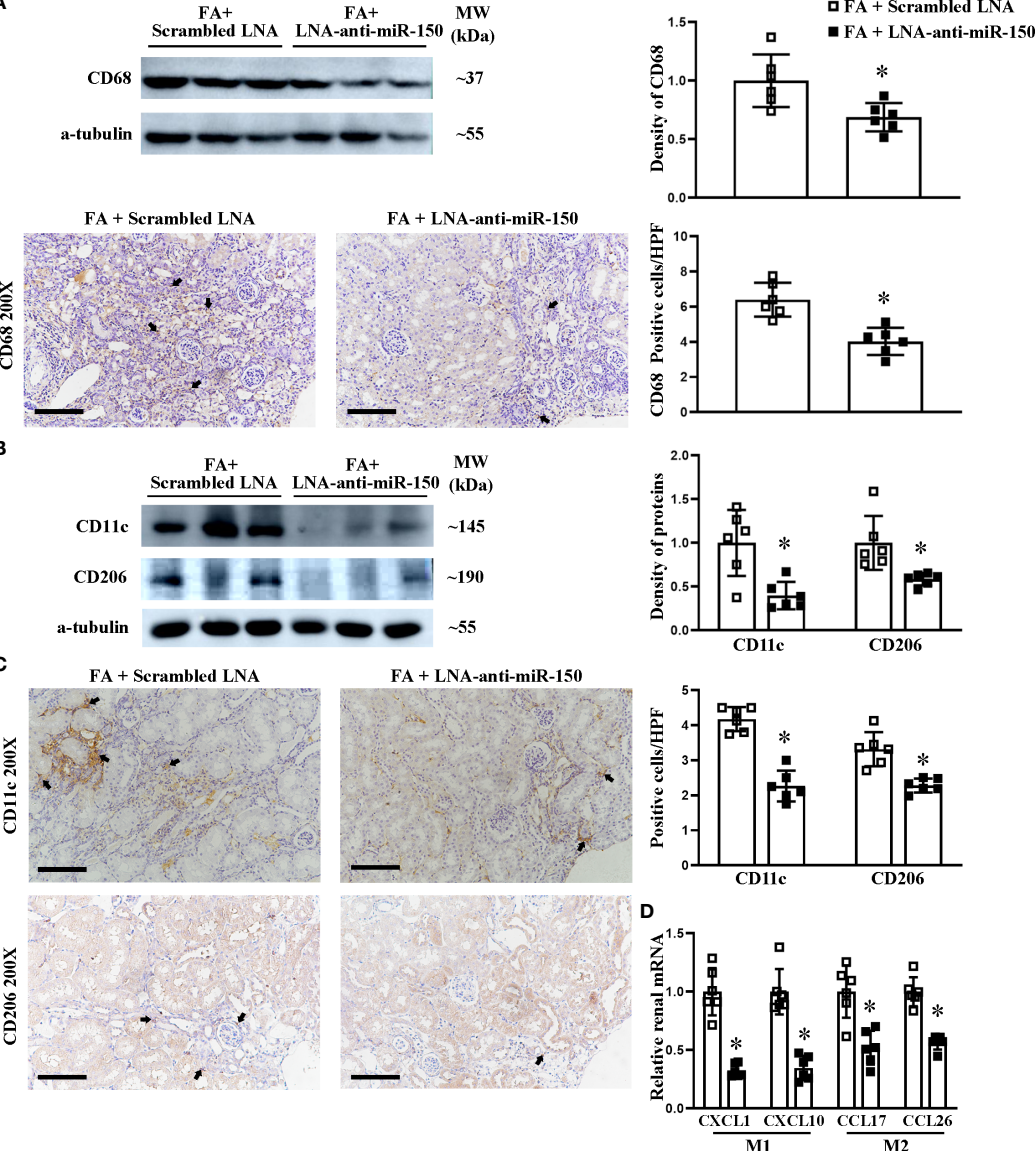
LNA-anti-miR-150 reduced the renal infiltration of macrophages and polarization of M1/M2 macrophages in renal interstitial fibrosis mice. Western blotting and immunohistochemical (IHC) staining of the renal expression of CD68 protein, a classic biomarker for total macrophages **(A)**. M1 macrophage polarization indicated by CD11c and M2 macrophage polarization indicated by CD206 expression as analyzed using Western blotting **(B)** and IHC staining **(C)**. The renal mRNA levels of M1-related cytokines CXCL1 and CXCL10 as well as M2-related cytokines CCL17 and CCL26 were quantitated by qPCR **(D)**. For IHC staining, magnification = ×200, scale bar = 100 μm. For all semi-quantification graphs, data are presented as mean ± SD, *n* = 6. *p < 0.05, FA + LNA-anti-miR-150 group vs. FA + scrambled LNA group.

Next, we investigated whether LNA-anti-miR-150 affects the polarization of macrophages to M1 and M2 phenotypes in folic acid-induced RIF mice. LNA-anti-miR-150 significantly decreased the renal protein levels of CD68, a macrophage marker, and reduced the numbers of CD68-expressing cells in folic acid-induced RIF mice, compared to the mice treated with the scrambled LNA, on Western blotting and immunohistochemical staining (**Figure 6A**).

We assessed the effect of LNA-anti-miR-150 on macrophage polarization. Using Western blotting of folic acid-induced RIF mouse kidneys, we found that LNA-anti-miR-150 reduced the CD11c+ M1 protein (**Figure 6B**). Similar findings were observed on immunohistochemical staining (**Figure 6C**). Similarly, the protein levels of CD206 and CD206+ M2 macrophages were decreased by the LNA-anti-miR-150 of folic acid-induced RIF mouse kidneys, respectively, on Western blotting (**Figure 6B**) and immunohistochemistry staining (**Figure 6C**). As for the macrophage-excreted cytokines, LNA-anti-miR-150 reduced M1-related CXCL1 and CXCL10 compared with the scrambled LNA-receiving RIF mouse kidneys. Similarly, M2 macrophage pro-inflammatory cytokines CCL17 and CCL26 were reduced in folic acid-induced RIF mouse kidneys by LNA-anti-miR-150 administration on qPCR (**Figure 6D**).

## Discussion

The main findings in this study of folic acid-induced renal fibrosis in mice are as follows: (1) LNA-anti-miR-150 ameliorated renal interstitial fibrosis at day 30 after the folic acid injection, (2) LNA-anti-miR-150 reduced the renal infiltration of total macrophages and pro-inflammatory polarized CD11c+ M1 and CD206+ M2 macrophages, and (3) LNA-anti-miR-150 regulated the protein expression of the SOCS1/JAK1/STAT1 pathway proteins.

Various mouse models, including unilateral ureteral obstruction and 5/6 nephrectomy, manifest renal fibrosis (18, 19). A bolus injection of folic acid provides the classic mouse model for the progression of AKI to RIF (20, 21). When renal function recovered, renal fibrosis still progressively and irreversibly occurred (22). In the present study, RIF was observed on morphological analysis at day 30 after a high-dose peritoneal injection of folic acid (**Figure 1**). Interestingly, we found that the expression of renal miR-150 was increased in folic acid-induced RIF mice. The increase of miR-150 in this model is consistent with our previous report (12). The overexpression of renal miR-150 accelerates the progression and renal fibrosis of murine lupus nephritis (14, 16, 23). In a clinical study of IgA nephropathy, Pawluczyk et al. reported that the expression of miR-150 was significantly increased (24). Similarly, Qi et al. found high levels of miR-150 in kidney tissue from patients with focal segmental glomerulosclerosis (15). Taken together, the clinical and translational studies suggest that miR-150 could be a promising therapeutic target in human glomerular diseases.

Chemically modified oligonucleotide small interfering RNAs and anti-miRs have been used to block the actions of specific endogenous genes and miRNA (25). More recently, LNA-anti-miR-132 has reduced liver fibrosis in a mouse model (26). Putta et al. reported that LNA-anti-miR-192-inhibited miR-192 levels attenuated glomerulosclerosis in diabetic mice (27). The treatment of *Trypanosoma cruzi*-infected mice with LNA-anti-miR-21 promoted a significant attenuation in cardiac fibrosis by inhibiting the effect of miR-21 on collagen production (28). miR-142-3p inhibitor reduced the tumorigenicity of breast cancer *in vitro* and *in vivo* (29). Our study found that LNA-anti-miR-150 strongly inhibited the renal endogenous miR-150 levels to lower than 30% of folic acid-induced interstitial fibrosis mice, which is similar to our previous study (12). Based on these data, we investigated the therapeutic effect of LNA-anti-miR-150 on interstitial fibrosis mice. LNA-anti-miR-150 alleviated renal interstitial fibrosis on PAS and Masson staining. LNA-anti-miR-150 also reduced the production of profibrotic proteins including a-SMA and FN (**Figure 2**). Dong et al. reported that silencing of miR-150 ameliorates diabetic nephropathy (30), and Qi et al. reported that miR-150 inhibitor ameliorates adriamycin-induced focal segmental glomerulosclerosis in mice (15). Ranganathan found that miR-150 deletion protected the kidneys from myocardial infarction-induced AKI in mice (31). Luan et al. also reported that miR-150-based RNA interference attenuates interstitial fibrosis in mice but did not characterize the mechanisms of protection (12). The present study is the first to explore the role of miRNA in protecting against folic acid-induced RIF mice.

It has been reported that miR-150 promotes renal fibrosis of lupus nephritis by downregulating the expression of SOCS1 in cultured kidney cells (16). miR-150 antagonist reversed the macrophage-induced decrease of SOCS1 and the increased JAK/STAT which is downstream of SOCS1 (12). The SOCS/JAK/STAT pathway has been involved in multiple kidney diseases, such as streptozotocin-induced diabetic nephropathy, ischemia–reperfusion-induced kidney injury, and cisplatin-induced AKI (32–34). However, whether the SOCS/JAK/STAT pathway participates in the pathogenesis of folic acid-induced RIF mice model remains unreported. In this study, we found that SOCS1 was downregulated and p-JAK1 and p-STAT1 were upregulated in folic acid-induced RIF mice. The renal expression changes of SOCS1, p-JAK1, and p-STAT1 were reversed to renal protective levels after eight doses of LNA-anti-miR-150 administration in 2 weeks (**Figures 3**, **Figure 4**). This data demonstrated that miR-150 was indeed an important gene regulating the SOCS1/JAK1/STAT1 pathway in folic acid-induced RIF mice. It is well established that miR-150 influences the development of immune cells. miR-150 is selectively expressed in mature B and T cells and is an important regulator for the differentiation and activation of B cells (35). In addition, KChIP-2, one of the miR-150 targets in T cells, has been reported to inhibit the production of IL-2, IL-4, and IFN-γ (36). Moreover, miR-150 has been reported to be involved in cytokine IL-17 expression (37). Our previous study mentioned that the inhibition of miR-150 in lupus nephritis can reduce the infiltration of total macrophages by just examining CD68, a total macrophage biomarker; we had not performed subsets of macrophages (14). There is not any report on the relationship between miR-150 and M1/M2 macrophages in kidney diseases.

In the present study, we focused on the role of pro-inflammatory CD11c+ M1 macrophages and CD206+ M2 macrophages. We found that renal total macrophages were upregulated. The CD11c+ M1 macrophages and CD206+ M2 macrophage numbers were also increased in folic acid-induced RIF mouse kidneys and located in the renal interstitial fibrosis area (**Figures 5C**, **6C**). These findings are consistent with prior reports (38). M1 macrophage polarization plays an important role in the progression of renal fibrosis due to the overproduction of pro-inflammatory cytokines and the profibrotic effect of pro-inflammatory cytokines (4). It is reasonable to propose that uncontrolled macrophage polarization might be an important underlying mechanism for the chronic inflammation and fibrosis observed in CKD (39, 40). Pro-inflammatory M1 macrophages induce renal injury from the early stages of the disease, and persistent existing M1-induced injury contributes to renal fibrosis in the late stages (4, 41).

The present study also showed that the CD206+ macrophage numbers are increased, as were the numbers of CD11c+ macrophages, at day 30 after FA-induced AKI when renal fibrosis appeared (**Figure 5**). These data suggest that M2 macrophages may also play crucial roles in the late stages of fibrosis, when epithelial–mesenchymal transition occurs. As supporting evidence, M2 macrophages contribute to fibrogenesis in the late stages of renal fibrosis, in part by producing transforming growth factor-beta, a potent pro-fibrotic cytokine (4). CD11b+/Ly6C^low^ M2 macrophages contribute to renal fibrosis by producing pro-fibrotic factors, including platelet-derived growth factor, insulin-like growth factor (IGF)-1, and CCL17, all of which are highly correlated with fibrogenesis or wound healing (42). In addition, using a single gene knockout, it was also shown that M2 macrophages express a secreted protein that is acidic and rich in cysteine (SPARC, also known as osteonectin), which regulates the production of extracellular matrix (ECM). Tissue inhibitor of metalloproteinase-2 (TIMP-2) prevents matrix metalloproteinase-mediated ECM turnover and thereby enhances matrix accumulation, contributing to cardiac fibrosis (43, 44). Furthermore, macrophage-derived IGF-1 attenuates myofibroblast apoptosis and enhances collagen production (45). In a mouse rhabdomyolysis-induced AKI model, macrophage polarization was also detected during disease progression. Abundant F4/80^low^CD11b^high^Ly6b^high^CD206^low^ macrophages are found in the kidney by day 2, whereas F4/80^high^CD11b+Ly6b^low^CD206^high^ cells become predominant by day 8 (46). When considering the published data and the findings presented here, it appears that both M1 and M2 macrophages play prominent roles in renal fibrosis.

LNA-anti-miR-150 decreased the infiltration of total macrophages and the polarized CD11c+ M1 macrophage and CD206+ M2 macrophage. In addition, the elevated secretion of pro-inflammatory cytokines, including M1-related CXCL1 and CXCL10 and M2-related CCL17 and CCL26, may augment in the early phase of renal inflammation and in the late phase of renal fibrosis in folic acid-induced RIF. The data suggests that LNA-anti-miR-150 alleviates the effect of CD11C+ M1 and CD206+ M2 on renal inflammation and fibrosis. What is the underlying mechanism? SOCS1 is a target of miR-150 (16). SOCS1 negatively regulates the JAK/STAT signaling pathway by binding JAKs or cytokine receptors (47). The downregulated SOCS1 expression activates the JAK1/STAT1 pathway and promotes the polarization of macrophages into M1 cells (48). The SOCS1/JAK/STAT pathway is involved in the role of M2 macrophage in cells and mice (49). However, the relationship between the SOCS1/JAK/STAT pathway and M2 macrophages in renal fibrosis remains unclear. In our study, we found that LNA-anti-miR-150 alleviates renal interstitial fibrosis by reducing pro-inflammatory CD11c+ M1 and CD206+ M2 macrophage polarization. Recently, different subtypes such as M2a, M2b, and M2c were identified (7). The diverse functions of different M2 subtypes in renal fibrosis merit future investigation.

In conclusion, LNA-anti-miR-150 alleviated mouse renal interstitial fibrosis induced by folic acid. The anti-renal fibrotic effects appear to be mediated by reducing the pro-inflammatory M1/M2 macrophage polarization regulated, at least partially, *via* the SOCS1/JAK1/STAT1 pathway.

## Data availability statement

The original contributions presented in the study are included in the article/**Supplementary Material**, further inquiries can be directed to the corresponding author/s.

## Ethics statement

The animal study was reviewed and approved by the Animal Care and Use Committee of China Medical University.

## Author contributions

HZ designed the studies and supervised the project. XH and JL performed most of the experiments and wrote the manuscript. JF, EL, JK, and JP revised the manuscript. CJ, CM, ZF, LZ, YZ, BZ, and YW performed the histological analysis. All authors contributed to the article and approved the submitted version.

## Funding

This research was supported by the Chinese Nature Science Foundation (81770698 and 82170740 to HZ and 82100743 to JL), the National Key R&D Program of China (SQ2017YFSF090121 to YZ), Liao Ning Revitalization Talents Program (XLYC2002081 to HZ), Key R&D Guidance Plan of Liaoning Province (2019JH8/10300009 to HZ), Pandeng Scholar of Liaoning Province (2013222 to HZ), and Outstanding Scientific Fund of Shengjing Hospital (HZ). JK is supported by the Intramural Research Program, NIDDK, NIH.

## Conflict of interest

The authors declare that the research was conducted in the absence of any commercial or financial relationships that could be construed as a potential conflict of interest.

## Publisher’s note

All claims expressed in this article are solely those of the authors and do not necessarily represent those of their affiliated organizations, or those of the publisher, the editors and the reviewers. Any product that may be evaluated in this article, or claim that may be made by its manufacturer, is not guaranteed or endorsed by the publisher.

## References

[1] Ruiz-Ortega M Lamas S Ortiz A. Antifibrotic agents for the management of CKD: A review. Am J Kidney Dis (2022) 80:251-263. doi: 10.1053/j.ajkd.2021.11.010 34999158

[2] Collaboration, G B D C K D. Global, regional, and national burden of chronic kidney disease, 1990-2017: a systematic analysis for the global burden of disease study 2017. Lancet (2020) 395:709–33. doi: 10.1016/S0140-6736(20)30045-3 PMC7049905 32061315

[3] Wen Y Yan HR Wang B Liu BC. Macrophage heterogeneity in kidney injury and fibrosis. Front Immunol (2021) 12:681748. doi: 10.3389/fimmu.2021.681748 34093584 PMC8173188

[4] Cao Q Harris DC Wang Y. Macrophages in kidney injury, inflammation, and fibrosis. Physiol (Bethesda) (2015) 30:183–94. doi: 10.1152/physiol.00046.2014 25933819

[5] Huen SC Cantley LG. Macrophages in renal injury and repair. Annu Rev Physiol (2017) 79:449–69. doi: 10.1146/annurev-physiol-022516-034219 28192060

[6] Engel JE Chade AR. Macrophage polarization in chronic kidney disease: a balancing act between renal recovery and decline? Am J Physiol Renal Physiol (2019) 317:F1409–f1413. doi: 10.1152/ajprenal.00380.2019 31566432 PMC6962510

[7] Tang PM Nikolic-Paterson DJ Lan HY. Macrophages: versatile players in renal inflammation and fibrosis. Nat Rev Nephrol (2019) 15:144–58. doi: 10.1038/s41581-019-0110-2 30692665

[8] Nelson MC O'Connell RM. MicroRNAs: At the interface of metabolic pathways and inflammatory responses by macrophages. Front Immunol (2020) 11:1797. doi: 10.3389/fimmu.2020.01797 32922393 PMC7456828

[9] Niu X Schulert GS. Functional regulation of macrophage phenotypes by MicroRNAs in inflammatory arthritis. Front Immunol (2019) 10:2217. doi: 10.3389/fimmu.2019.02217 31572403 PMC6753331

[10] Ding C Zheng J Wang B Li Y Xiang H Dou M. Exosomal MicroRNA-374b-5p from tubular epithelial cells promoted M1 macrophages activation and worsened renal Ischemia/Reperfusion injury. Front Cell Dev Biol (2020) 8:587693. doi: 10.3389/fcell.2020.587693 33324643 PMC7726230

[11] Zhang Y Cai S Ding X Lu C Wu R Wu H. MicroRNA-30a-5p silencing polarizes macrophages toward M2 phenotype to alleviate cardiac injury following viral myocarditis by targeting SOCS1. Am J Physiol Heart Circ Physiol (2021) 320:H1348–h1360. doi: 10.1152/ajpheart.00431.2020 33416455

[12] Luan J Fu J Wang D Jiao C Cui X Chen C. miR-150-Based RNA interference attenuates tubulointerstitial fibrosis through the SOCS1/JAK/STAT pathway *In vivo* and in vitro. Mol Ther Nucleic Acids (2020) 22:871–84. doi: 10.1016/j.omtn.2020.10.008 PMC7658580 33230482

[13] Zhou H Miyaji T Kato A Fujigaki Y Sano K Hishida A. Attenuation of cisplatin-induced acute renal failure is associated with less apoptotic cell death. J Lab Clin Med (1999) 134:649–58. doi: 10.1016/s0022-2143(99)90106-3 10595794

[14] Luan J Fu J Chen C Jiao C Kong W Zhang Y. LNA-anti-miR-150 ameliorated kidney injury of lupus nephritis by inhibiting renal fibrosis and macrophage infiltration. Arthritis Res Ther (2019) 21:276. doi: 10.1186/s13075-019-2044-2 31829247 PMC6907329

[15] Qi H Fu J Luan J Jiao C Cui X Cao X. miR-150 inhibitor ameliorates adriamycin-induced focal segmental glomerulosclerosis. Biochem Biophys Res Commun (2020) 522:618–25. doi: 10.1016/j.bbrc.2019.11.096 31787235

[16] Zhou H Hasni SA Perez P Tandon M Jang SI Zheng C. miR-150 promotes renal fibrosis in lupus nephritis by downregulating SOCS1. J Am Soc Nephrol (2013) 24:1073–87. doi: 10.1681/asn.2012080849 PMC3699828 23723424

[17] Li H Watowich S. Innate immune regulation by STAT-mediated transcriptional mechanisms. Immunol Rev (2014) 261:84–101. doi: 10.1111/imr.12198 25123278 PMC4135451

[18] Li H Duann P Li Z Zhou X Ma J Rovin BH. The cell membrane repair protein MG53 modulates transcription factor NF-κB signaling to control kidney fibrosis. Kidney Int (2022) 101:119–30. doi: 10.1016/j.kint.2021.09.027 PMC8741748 34757120

[19] Shi J Yang Y Wang YN Li Q Xing X Cheng AY. Oxidative phosphorylation promotes vascular calcification in chronic kidney disease. Cell Death Dis (2022) 13:229. doi: 10.1038/s41419-022-04679-y 35277475 PMC8917188

[20] Martin-Sanchez D Guerrero-Mauvecin J Fontecha-Barriuso M Mendez-Barbero N Saiz ML Lopez-Diaz AM. Bone marrow-derived RIPK3 mediates kidney inflammation in acute kidney injury. J Am Soc Nephrol (2022) 33:357–73. doi: 10.1681/asn.2021030383 PMC8819996 35046131

[21] Ajay AK Zhao L Vig S Fujiwara M Thakurela S Jadhav S. Deletion of STAT3 from Foxd1 cell population protects mice from kidney fibrosis by inhibiting pericytes trans-differentiation and migration. Cell Rep (2022) 38:110473. doi: 10.1016/j.celrep.2022.110473 35263586 PMC10027389

[22] Li R Guo Y Zhang Y Zhang X Zhu L Yan T. Salidroside ameliorates renal interstitial fibrosis by inhibiting the TLR4/NF-κB and MAPK signaling pathways. Int J Mol Sci (2019) 20:1103–18. doi: 10.3390/ijms20051103 PMC6429495 30836660

[23] Luan J Jiao C Kong W Fu J Qu W Chen Y. circHLA-c plays an important role in lupus nephritis by sponging miR-150. Mol Ther Nucleic Acids (2018) 10:245–53. doi: 10.1016/j.omtn.2017.12.006 PMC5768151 29499937

[24] Pawluczyk IZA Didangelos A Barbour SJ Er L Becker JU Martin R. Differential expression of microRNA miR-150-5p in IgA nephropathy as a potential mediator and marker of disease progression. Kidney Int (2021) 99:1127–39. doi: 10.1016/j.kint.2020.12.028 33417998

[25] Dhuri K Gaddam RR Vikram A Slack FJ Bahal R. Therapeutic potential of chemically modified, synthetic, triplex peptide nucleic acid-based oncomir inhibitors for cancer therapy. Cancer Res (2021) 81:5613–24. doi: 10.1158/0008-5472.can-21-0736 PMC8595710 34548334

[26] Momen-Heravi F Catalano D Talis A Szabo G Bala S. Protective effect of LNA-anti-miR-132 therapy on liver fibrosis in mice. Mol Ther Nucleic Acids (2021) 25:155–67. doi: 10.1016/j.omtn.2021.05.007 PMC8368790 34458001

[27] Putta S Lanting L Sun G Lawson G Kato M Natarajan R. Inhibiting microRNA-192 ameliorates renal fibrosis in diabetic nephropathy. J Am Soc Nephrol (2012) 23:458–69. doi: 10.1681/asn.2011050485 PMC3294315 22223877

[28] Nonaka CKV Sampaio GL Silva KN Khouri R Macedo CT Chagas Translational Research, C. Therapeutic miR-21 silencing reduces cardiac fibrosis and modulates inflammatory response in chronic chagas disease. Int J Mol Sci (2021) 22:3307-3325. doi: 10.3390/ijms22073307 33804922 PMC8036348

[29] Naseri Z Oskuee RK Jaafari MR Forouzandeh Moghadam, M. Exosome-mediated delivery of functionally active miRNA-142-3p inhibitor reduces tumorigenicity of breast cancer *in vitro* and *in vivo* . Int J Nanomedicine (2018) 13:7727–47. doi: 10.2147/ijn.s182384 PMC6251455 30538455

[30] Dong W Zhang H Zhao C Luo Y Chen Y. Silencing of miR-150-5p ameliorates diabetic nephropathy by targeting SIRT1/p53/AMPK pathway. Front Physiol (2021) 12:624989. doi: 10.3389/fphys.2021.624989 33897448 PMC8064124

[31] Ranganathan P Jayakumar C Tang Y Park KM Teoh JP Su H. MicroRNA-150 deletion in mice protects kidney from myocardial infarction-induced acute kidney injury. Am J Physiol Renal Physiol (2015) 309:F551–558. doi: 10.1152/ajprenal.00076.2015 PMC4572391 26109086

[32] Zhu M Wang H Chen J Zhu H. Sinomenine improve diabetic nephropathy by inhibiting fibrosis and regulating the JAK2/STAT3/SOCS1 pathway in streptozotocin-induced diabetic rats. Life Sci (2021) 265:118855. doi: 10.1016/j.lfs.2020.118855 33278392

[33] Susnik N Sörensen-Zender I Rong S von Vietinghoff S Lu X Rubera I. Ablation of proximal tubular suppressor of cytokine signaling 3 enhances tubular cell cycling and modifies macrophage phenotype during acute kidney injury. Kidney Int (2014) 85:1357–68. doi: 10.1038/ki.2013.525 24402091

[34] Tsogbadrakh B Ryu H Ju KD Lee J Yun S Yu KS. AICAR, an AMPK activator, protects against cisplatin-induced acute kidney injury through the JAK/STAT/SOCS pathway. Biochem Biophys Res Commun (2019) 509:680–6. doi: 10.1016/j.bbrc.2018.12.159 30616891

[35] Zhou B Wang S Mayr C Bartel DP Lodish HF. miR-150, a microRNA expressed in mature b and T cells, blocks early b cell development when expressed prematurely. Proc Natl Acad Sci U.S.A. (2007) 104:7080–5. doi: 10.1073/pnas.0702409104 PMC1855395 17438277

[36] Savignac M Pintado B Gutierrez-Adan A Palczewska M Mellström B Naranjo JR. Transcriptional repressor DREAM regulates T-lymphocyte proliferation and cytokine gene expression. EMBO J (2005) 24:3555–64. doi: 10.1038/sj.emboj.7600810 PMC1276700 16177826

[37] Hu Z Cui Y Qiao X He X Li F Luo C. Silencing miR-150 ameliorates experimental autoimmune encephalomyelitis. Front Neurosci (2018) 12:465. doi: 10.3389/fnins.2018.00465 30050402 PMC6052910

[38] Haruhara K Suzuki T Wakui H Azushima K Kurotaki D Kawase W. Deficiency of the kidney tubular angiotensin II type1 receptor-associated protein ATRAP exacerbates streptozotocin-induced diabetic glomerular injury *via* reducing protective macrophage polarization. Kidney Int (2022) 101:912–28. doi: 10.1016/j.kint.2022.01.031 35240129

[39] Li C Ding X Xiang D Xu J Huang X Hou F. Enhanced M1 and impaired M2 macrophage polarization and reduced mitochondrial biogenesis *via* inhibition of AMP kinase in chronic kidney disease. Cell Physiol Biochem (2015) 36:358–72. doi: 10.1159/000430106 25967974

[40] Tang P Zhang Y Xiao J Tang P Chung J Li J. Neural transcription factor Pou4f1 promotes renal fibrosis *via* macrophage-myofibroblast transition. Proc Natl Acad Sci U.S.A. (2020) 117:20741–52. doi: 10.1073/pnas.1917663117 PMC7456094 32788346

[41] Wen Y Lu X Ren J Privratsky J Yang B Rudemiller N. αKLF4 in macrophages attenuates TNF-mediated kidney injury and fibrosis. J Am Soc Nephrol (2019) 30:1925–38. doi: 10.1681/asn.2019020111 PMC6779357 31337692

[42] Duffield JS. Macrophages and immunologic inflammation of the kidney. Semin Nephrol (2010) 30:234–54. doi: 10.1016/j.semnephrol.2010.03.003 PMC2922007 20620669

[43] Fan D Takawale A Basu R Patel V Lee J Kandalam V. Differential role of TIMP2 and TIMP3 in cardiac hypertrophy, fibrosis, and diastolic dysfunction. Cardiovasc Res (2014) 103:268–80. doi: 10.1093/cvr/cvu072 24692173

[44] Wang JC Lai S Guo X Zhang X de Crombrugghe B Sonnylal S. Attenuation of fibrosis *in vitro* and *in vivo* with SPARC siRNA. Arthritis Res Ther (2010) 12:R60. doi: 10.1186/ar2973 20359365 PMC2888211

[45] Wynes MW Frankel SK Riches DW. IL-4-induced macrophage-derived IGF-I protects myofibroblasts from apoptosis following growth factor withdrawal. J Leukoc Biol (2004) 76:1019–27. doi: 10.1189/jlb.0504288 15316031

[46] Belliere J Casemayou A Ducasse L Zakaroff-Girard A Martins F Iacovoni JS. Specific macrophage subtypes influence the progression of rhabdomyolysis-induced kidney injury. J Am Soc Nephrol (2015) 26:1363–77. doi: 10.1681/asn.2014040320 PMC4446873 25270069

[47] Yoshimura A Naka T Kubo M. SOCS proteins, cytokine signalling and immune regulation. Nat Rev Immunol (2007) 7:454–65. doi: 10.1038/nri2093 17525754

[48] Liang Y Tang H Chen Z Zeng L Wu J Yang W. Downregulated SOCS1 expression activates the JAK1/STAT1 pathway and promotes polarization of macrophages into M1 type. Mol Med Rep (2017) 16:6405–11. doi: 10.3892/mmr.2017.7384 28901399

[49] Whyte CS Bishop ET Rückerl D Gaspar-Pereira S Barker RN Allen JE. Suppressor of cytokine signaling (SOCS)1 is a key determinant of differential macrophage activation and function. J Leukoc Biol (2011) 90:845–54. doi: 10.1189/jlb.1110644 21628332

