# Supplementary material for: LNA-anti-miR-150 alleviates renal interstitial fibrosis by reducing pro-inflammatory M1/M2 macrophage polarization
Source: Front Immunol. 2022 Aug 5;13:913007. doi: 10.3389/fimmu.2022.913007 (PMC9389080; doi:10.3389/fimmu.2022.913007)
Supplement: Supplementary file 1 [file DataSheet_1.pdf]

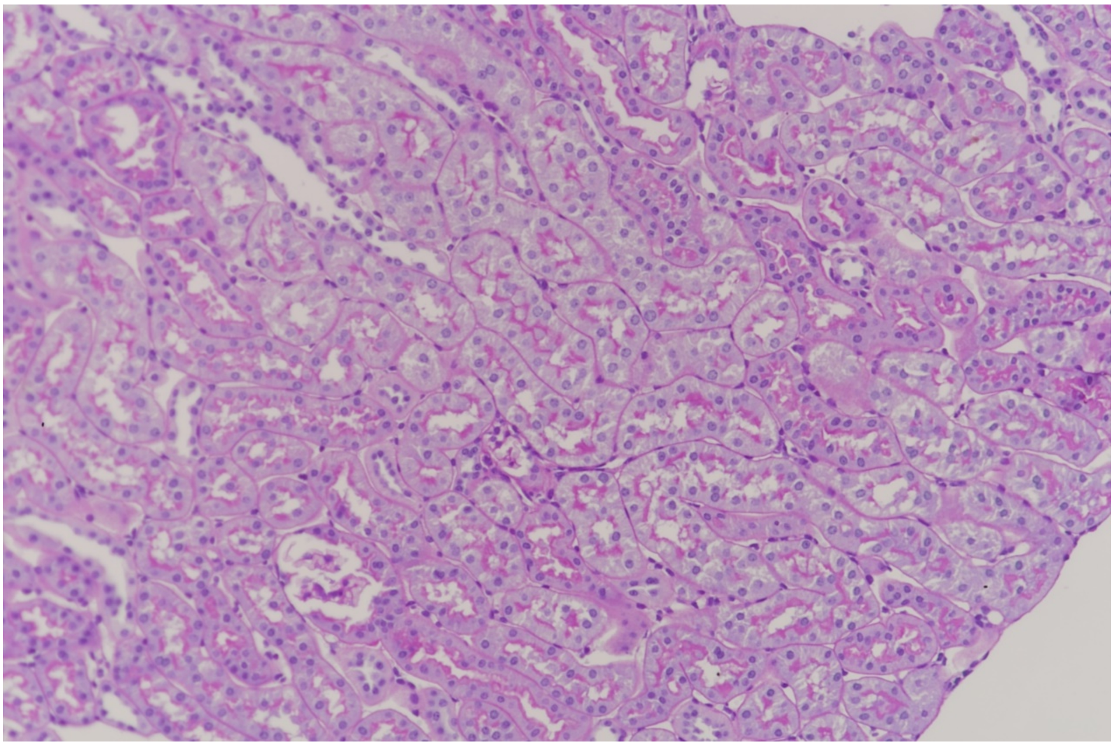

Fig 1C day0 for PAS

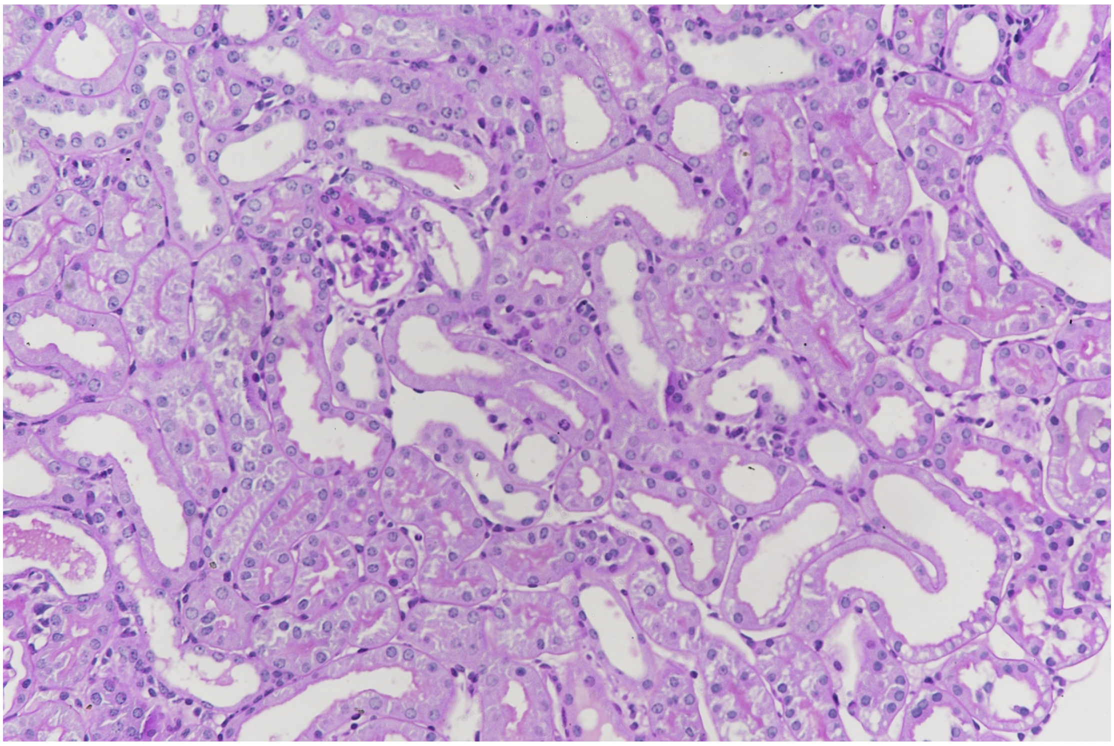

Fig 1C day2 for PAS

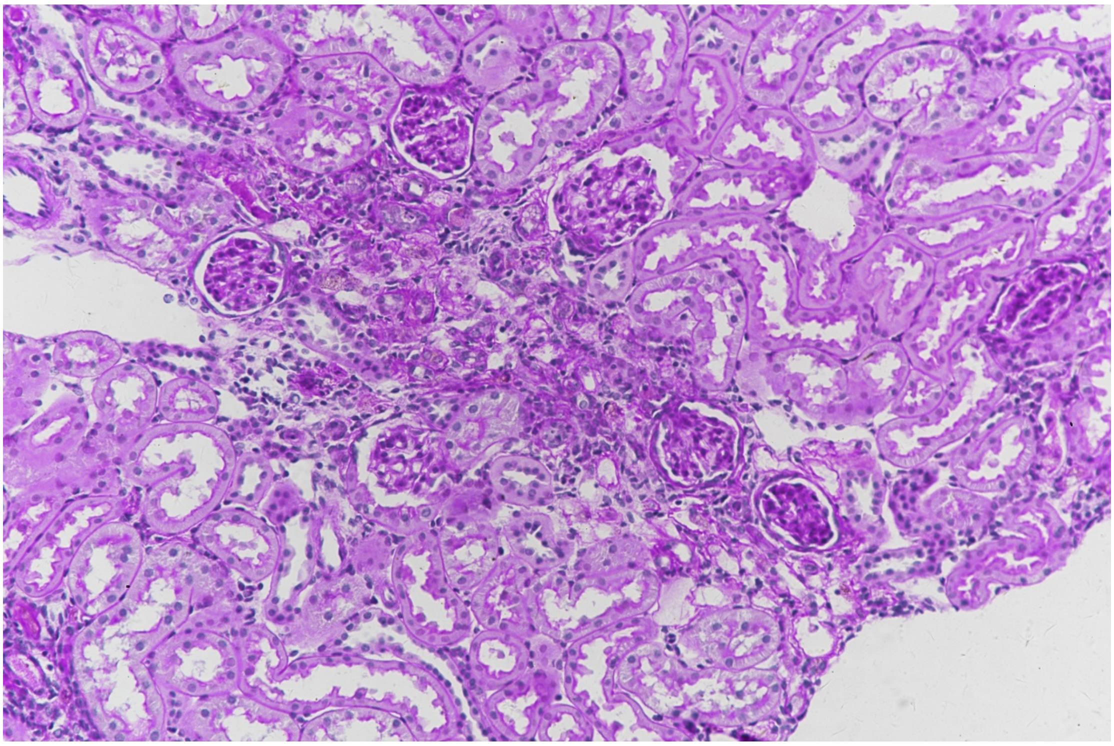

Fig 1C day30 for PAS

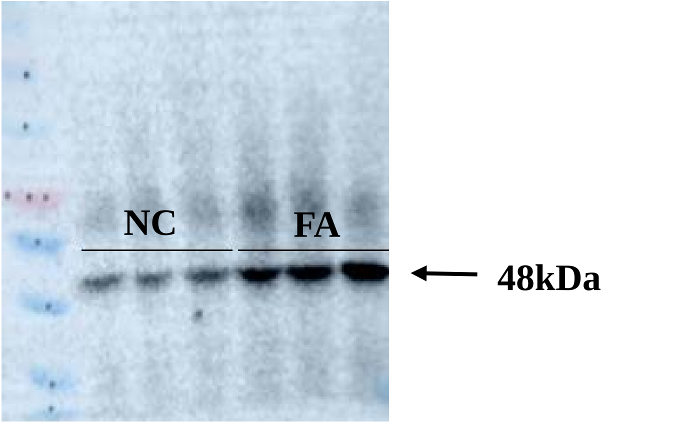

Fig 1D for a-SMA

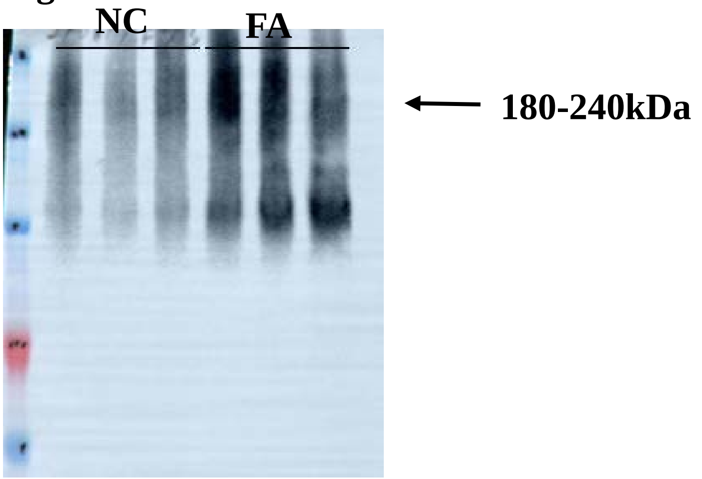

Fig 1D for FN

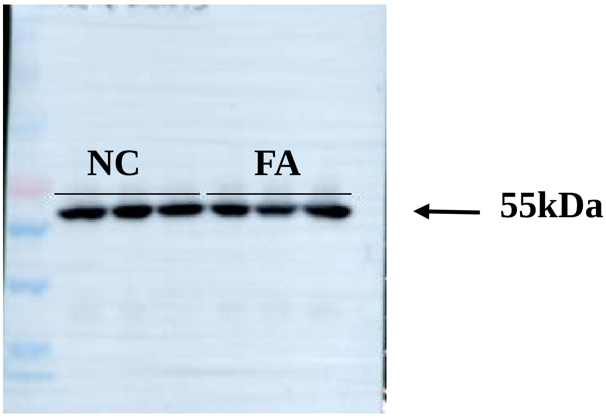

Fig 1D for a-tubulin

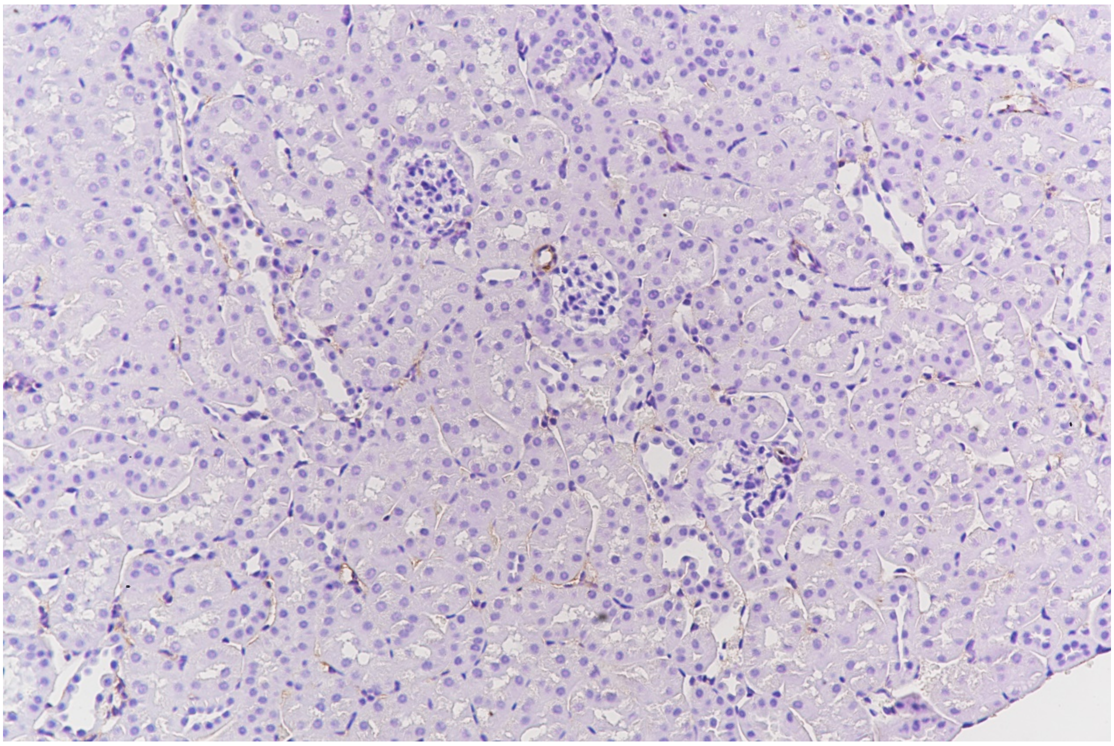

Fig 1E NC for a-SMA

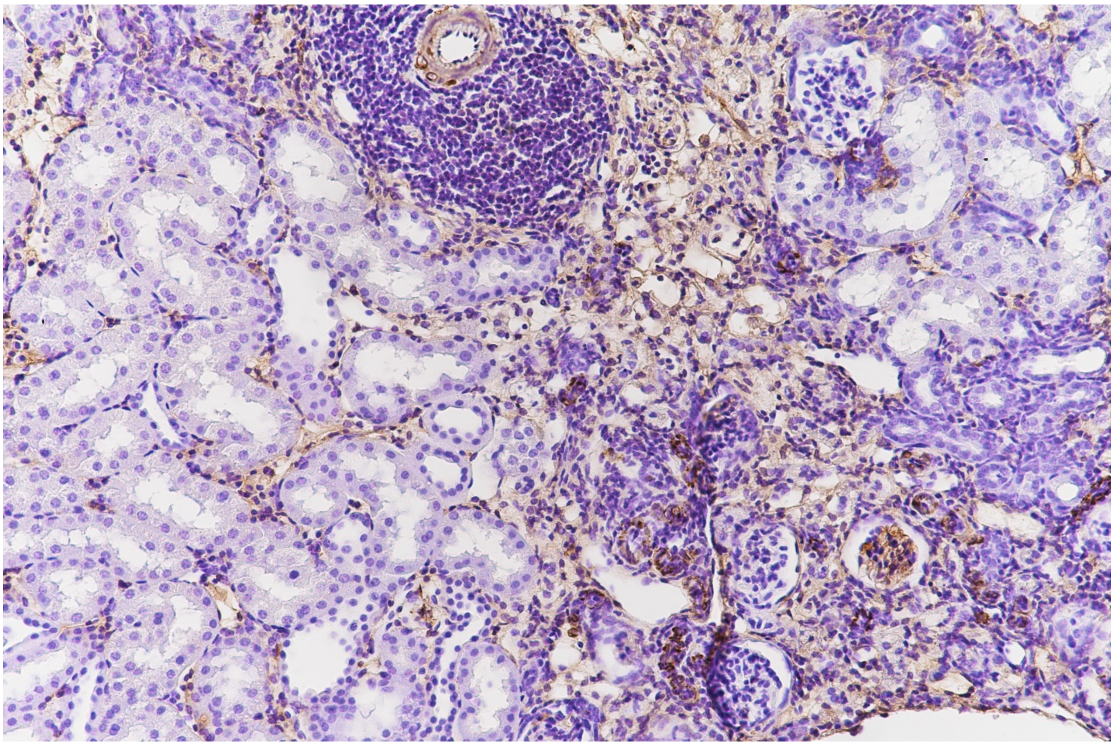

Fig 1E FA for a-SMA

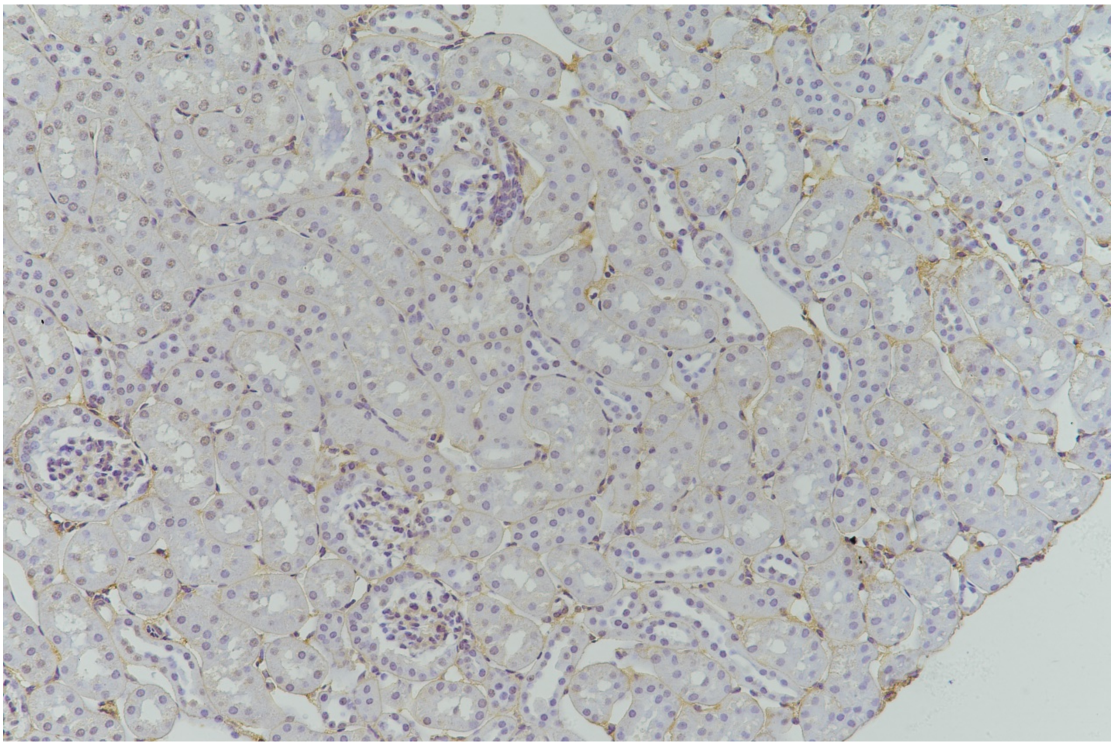

Fig 1E NC for FN

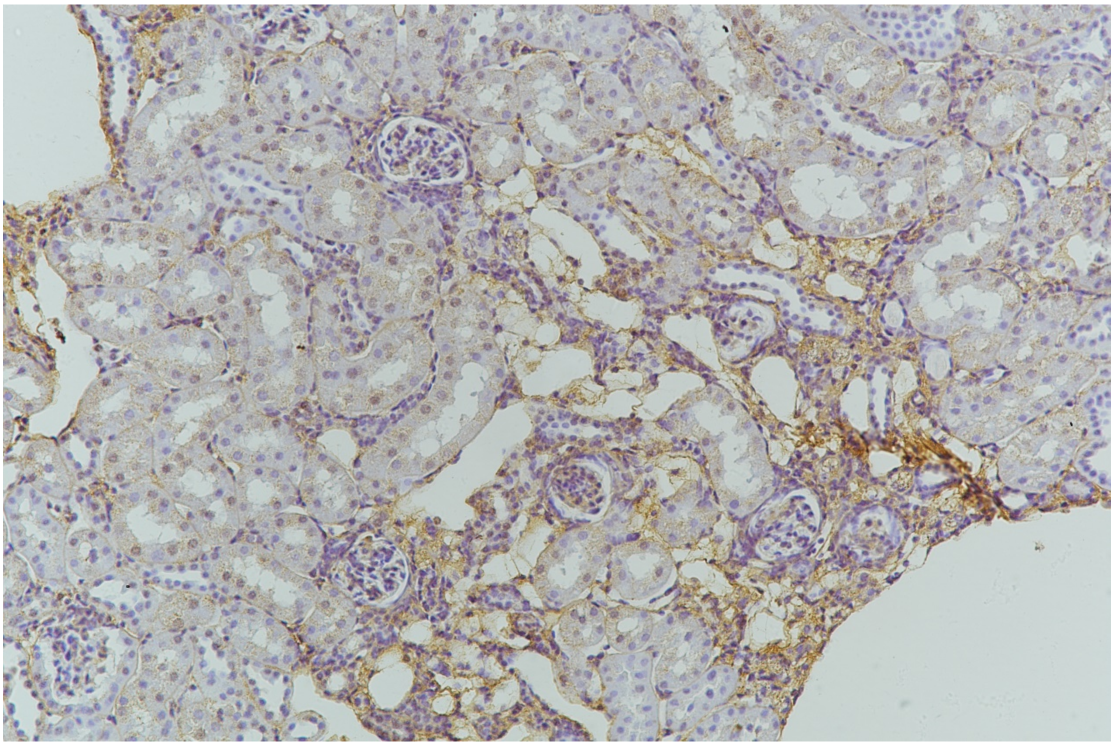

Fig 1E FA for FN

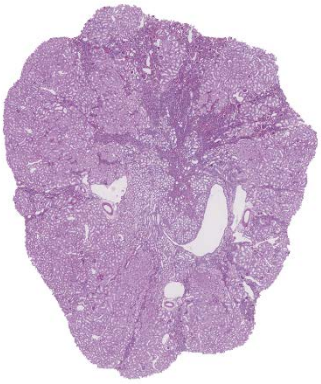

Fig 2B FA + Scrambled LNA for PAS (1.5X)

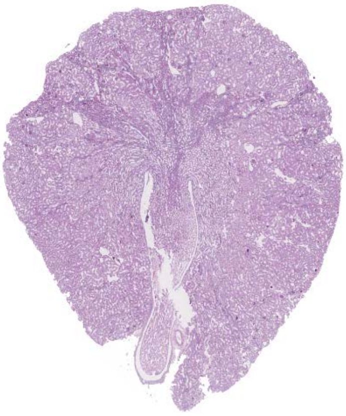

Fig 2B FA + LNA-anti-miR-150 for PAS (1.5X)

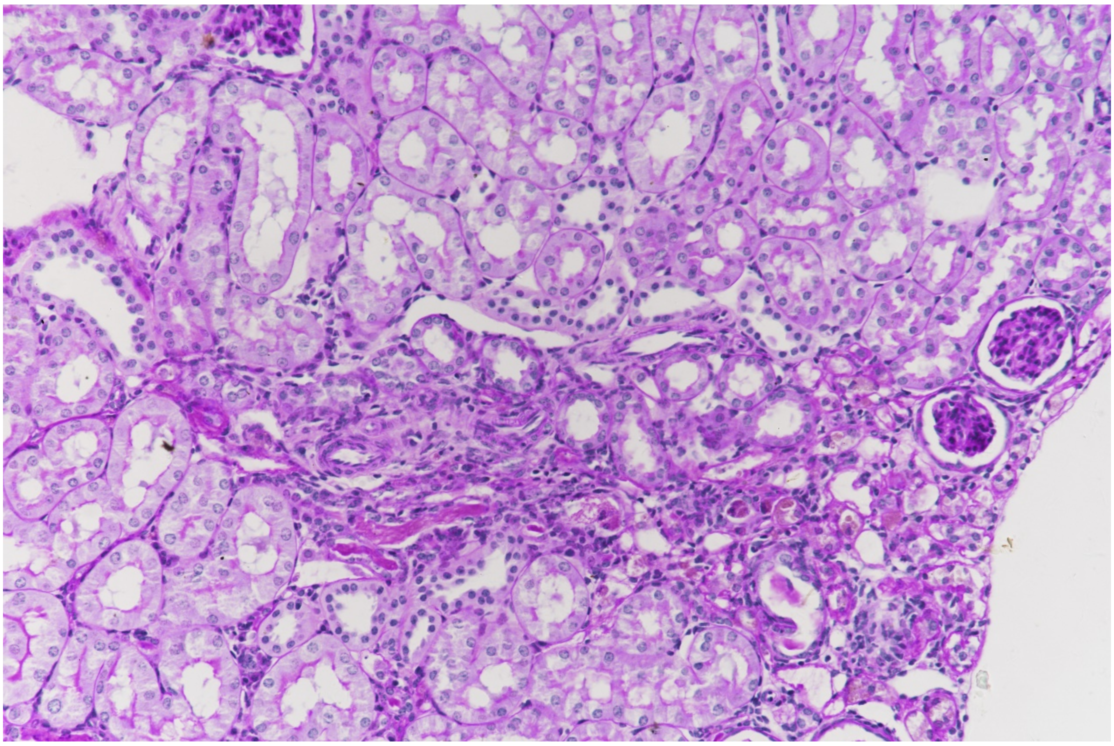

Fig 2C FA + Scrambled LNA for PAS (200X)

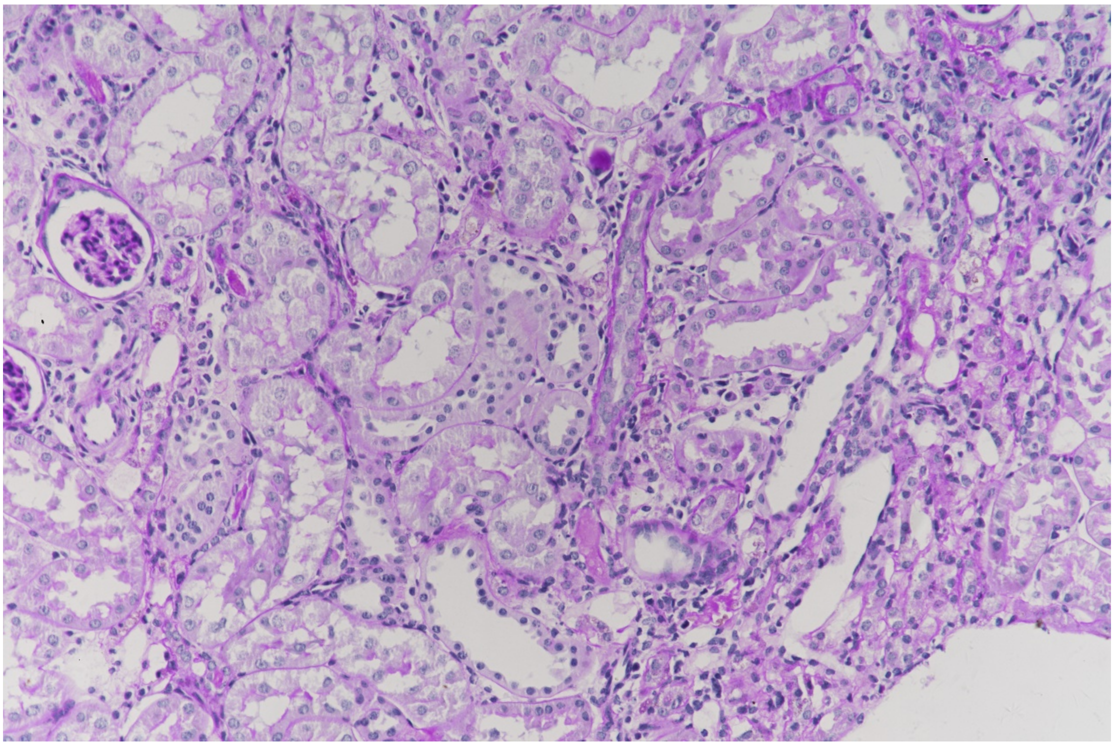

Fig 2C FA + LNA-anti-miR-150 for PAS (200X)

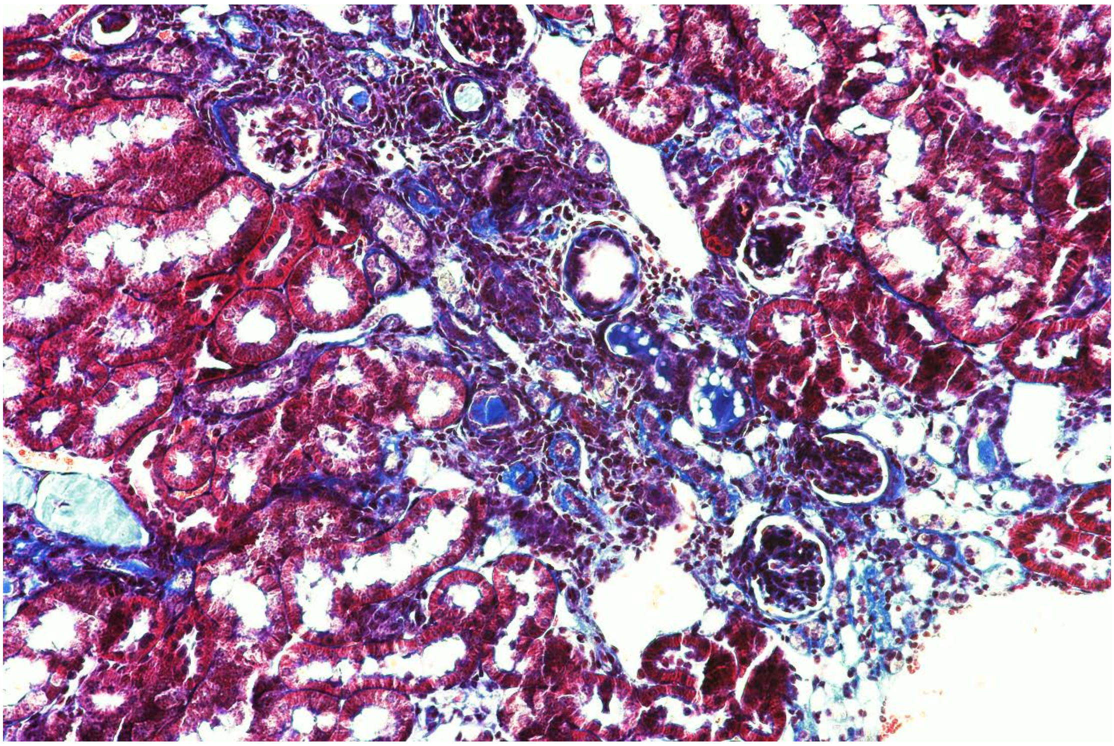

Fig 2D FA + Scrambled LNA for MASSON

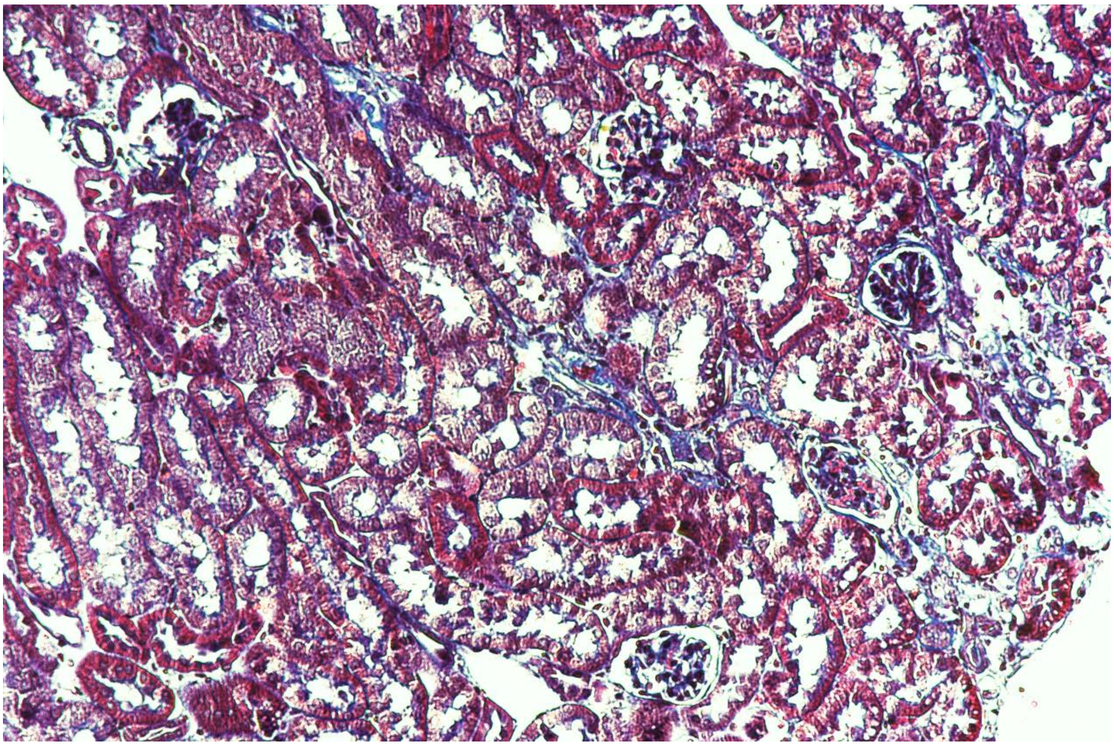

Fig 2D FA + LNA-anti-miR-150 for MASSON

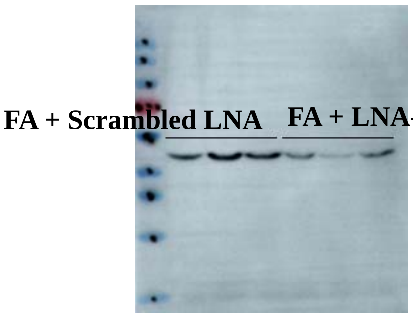

Fig 2E for a-SMA

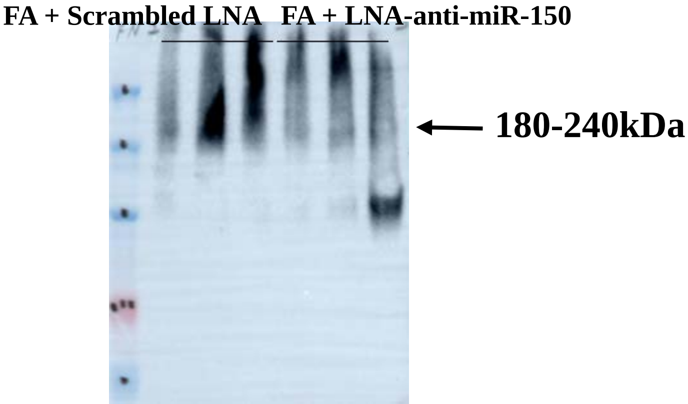

Fig 2E for FN

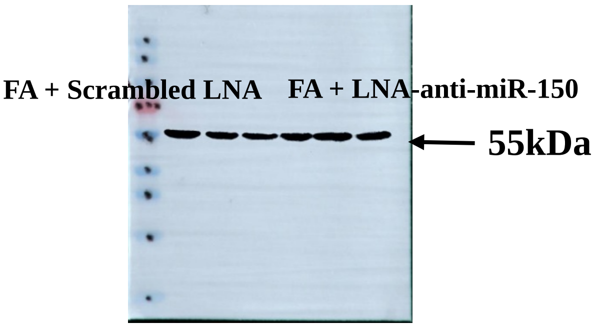

Fig 2E for a-tubulin

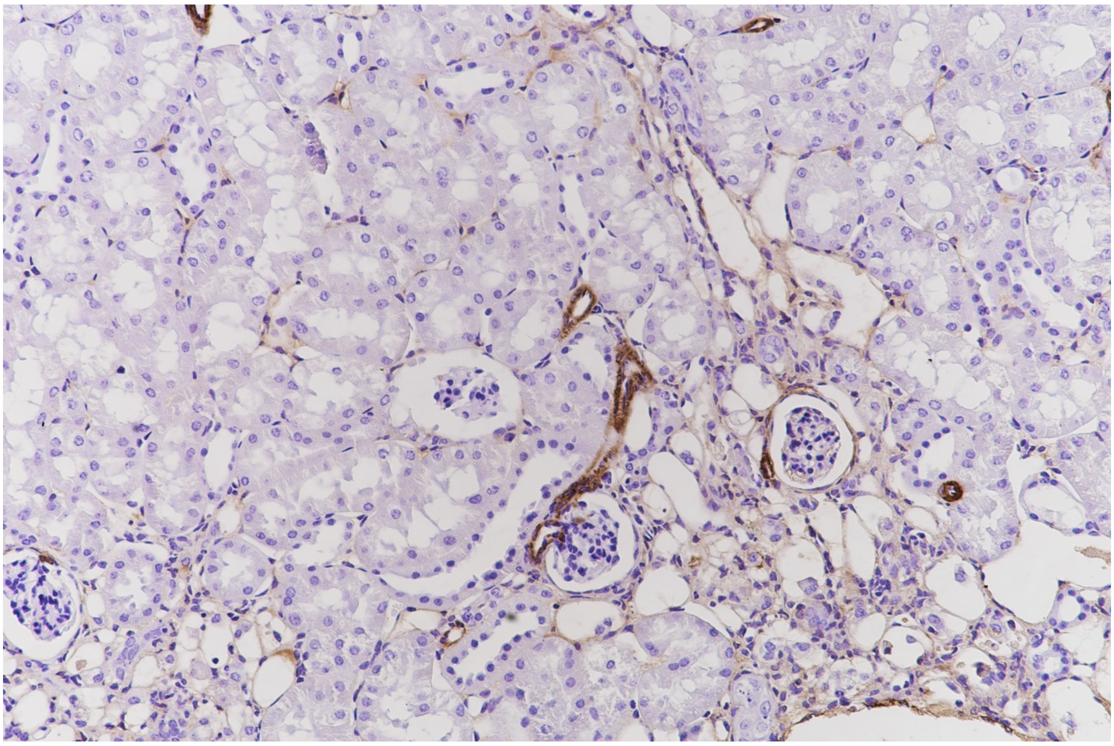

Fig 2F FA + Scrambled LNA for a-SMA

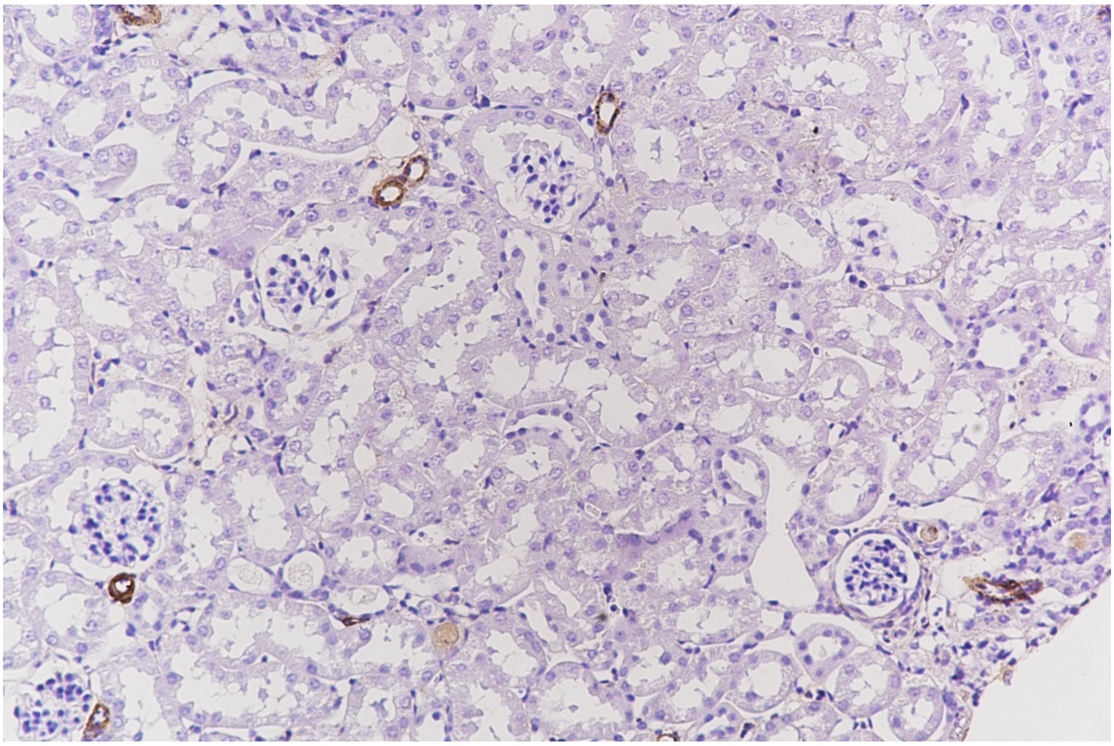

Fig 2F FA + LNA-anti-miR-150 for a-SMA

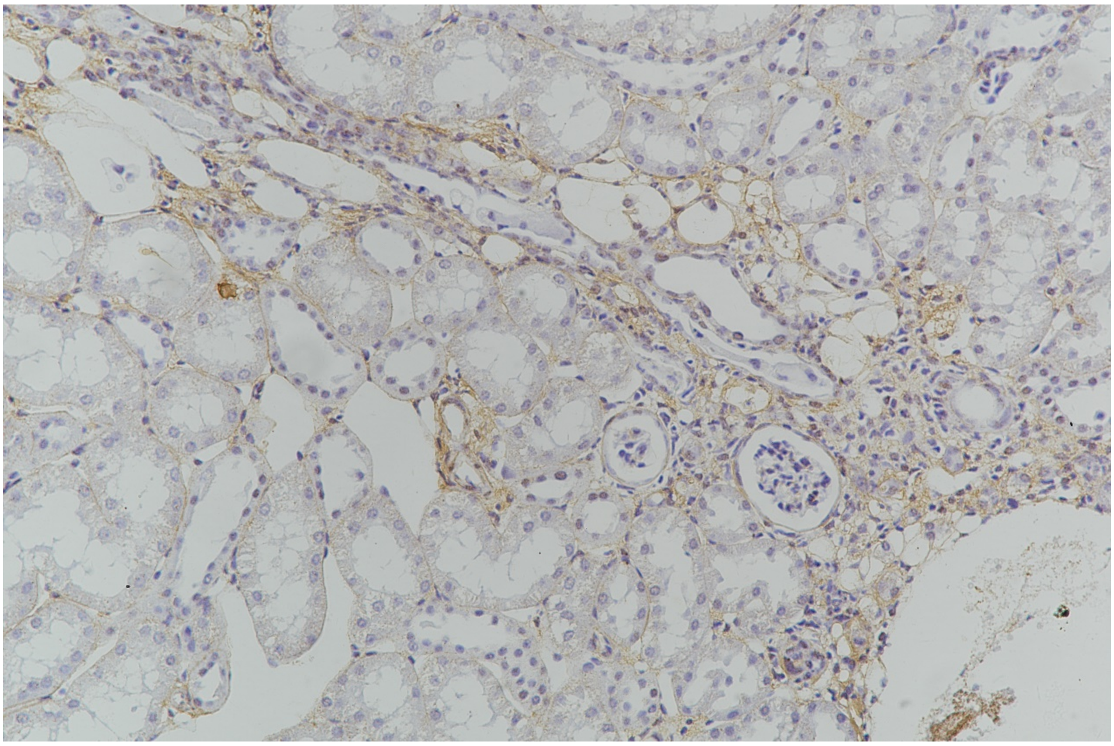

Fig 2F FA + Scrambled LNA for FN

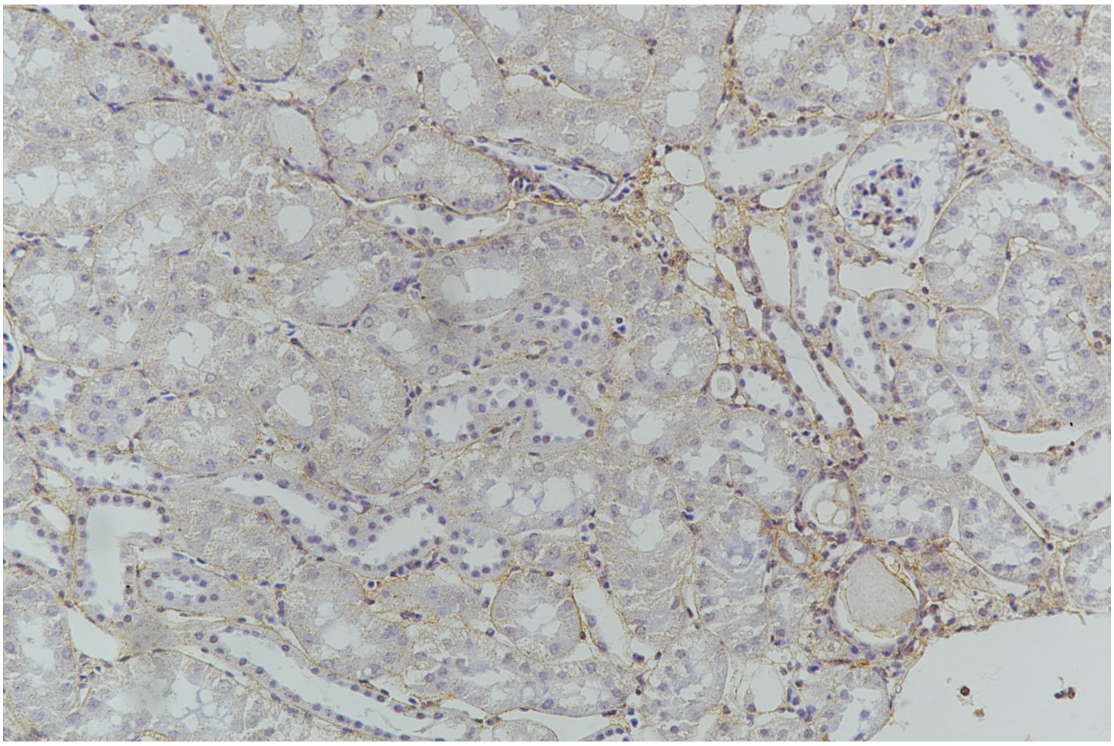

Fig 2F FA + LNA-anti-miR-150 for FN

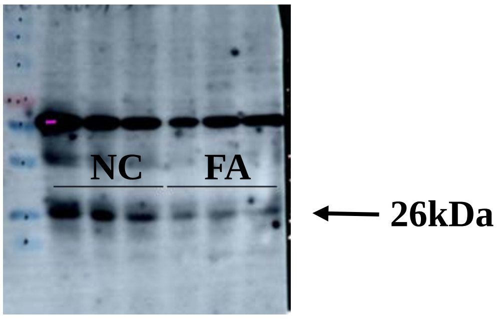

Fig 3A for SOCS1

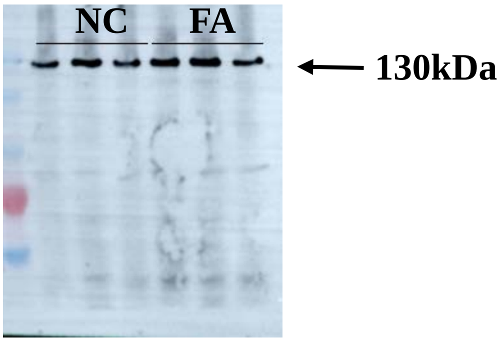

Fig 3A for JAK1

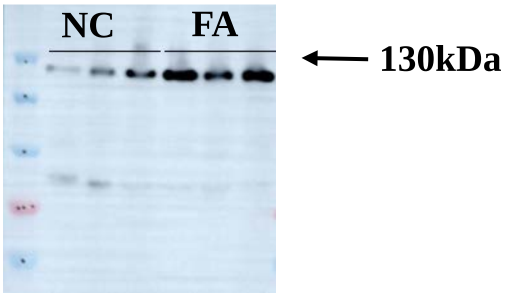

Fig 3A for p-JAK1

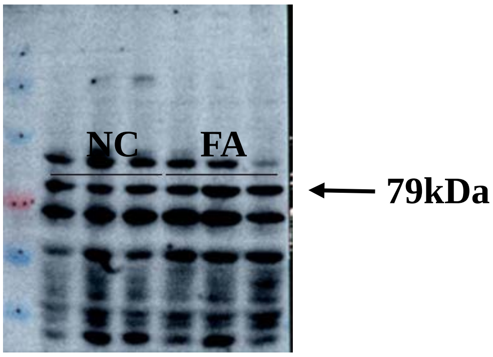

Fig 3A for STAT1

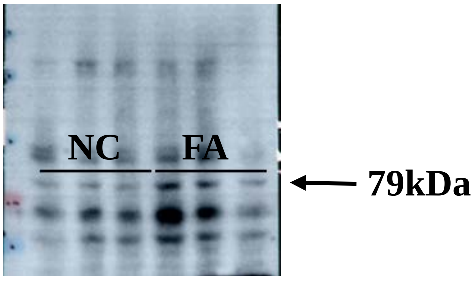

Fig 3A for p-STAT1

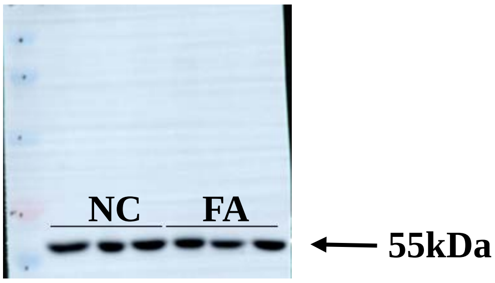

Fig 3A for a-tubulin

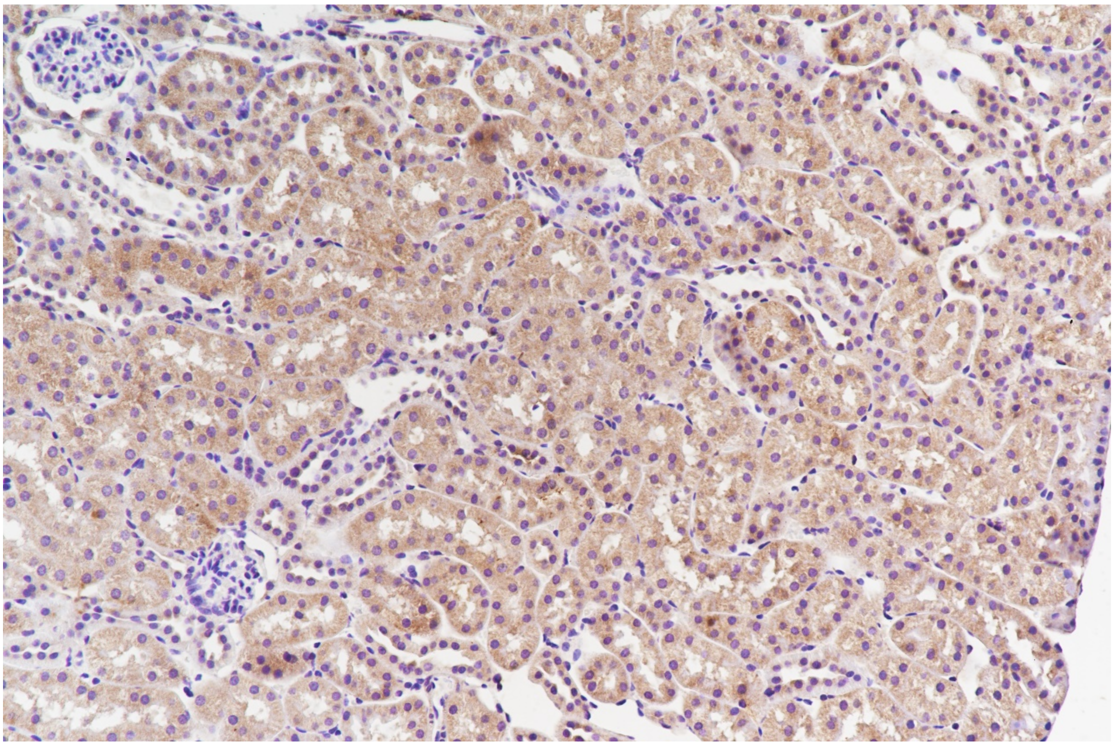

Fig 3B NC for SOCS1

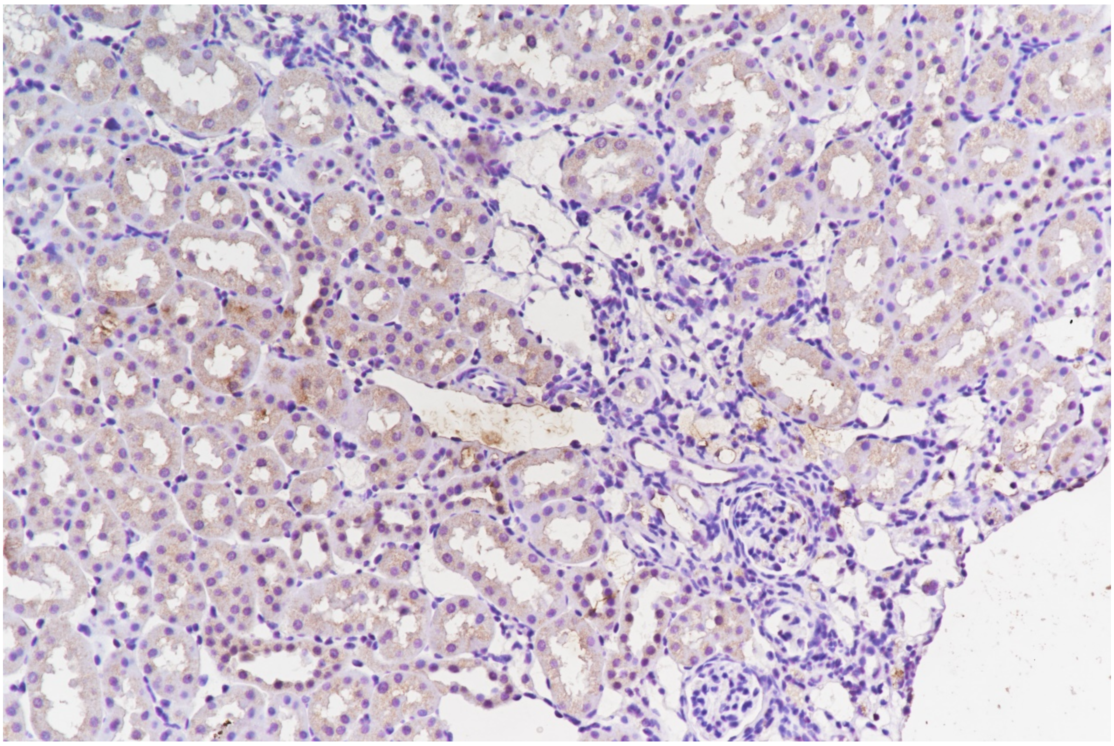

Fig 3B FA for SOCS1

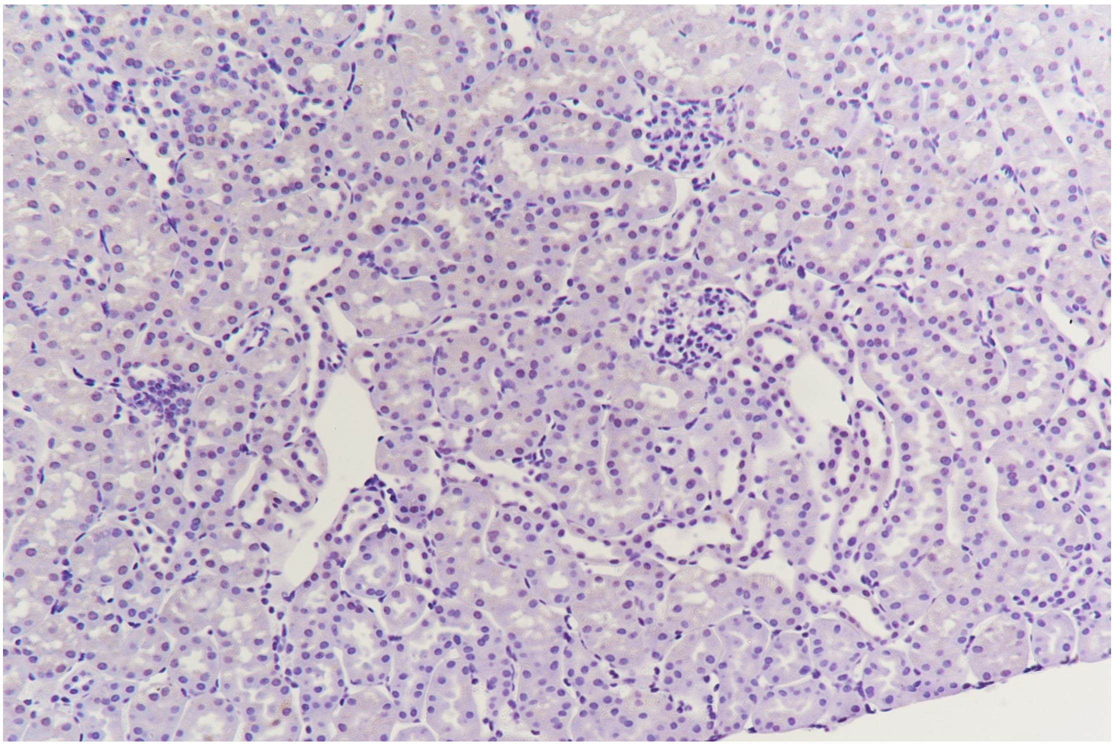

Fig 3B NC for p-JAK1

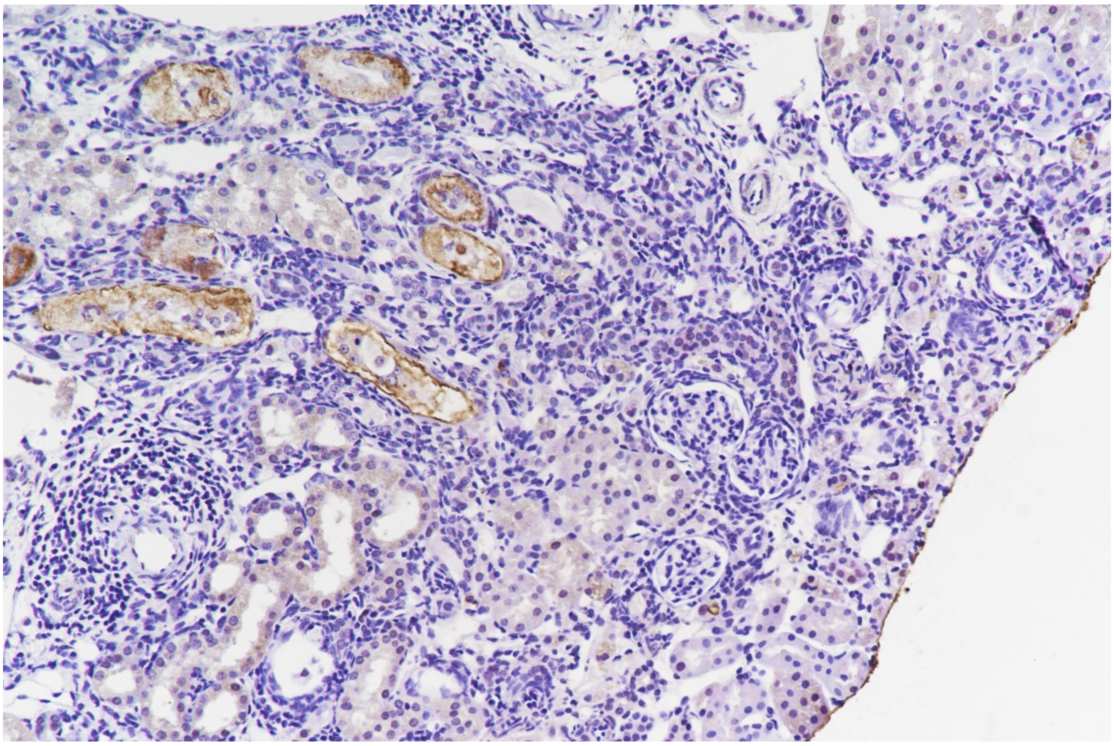

Fig 3B FA for p-JAK1

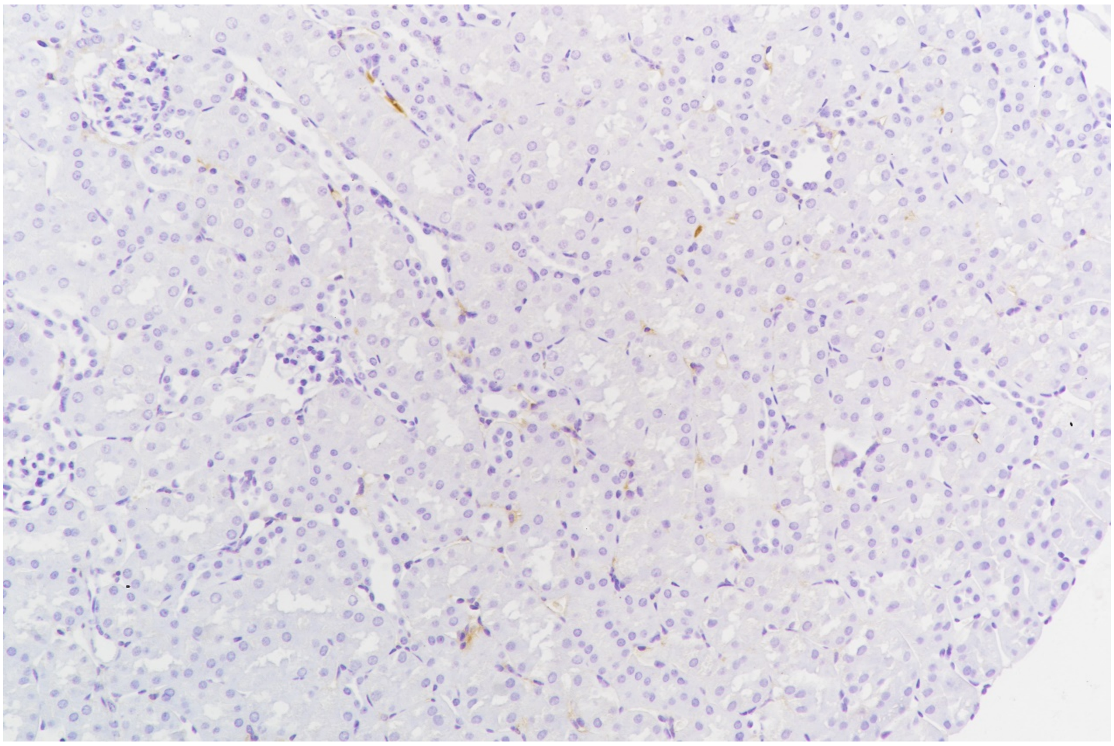

Fig 3B NC for p-STAT1

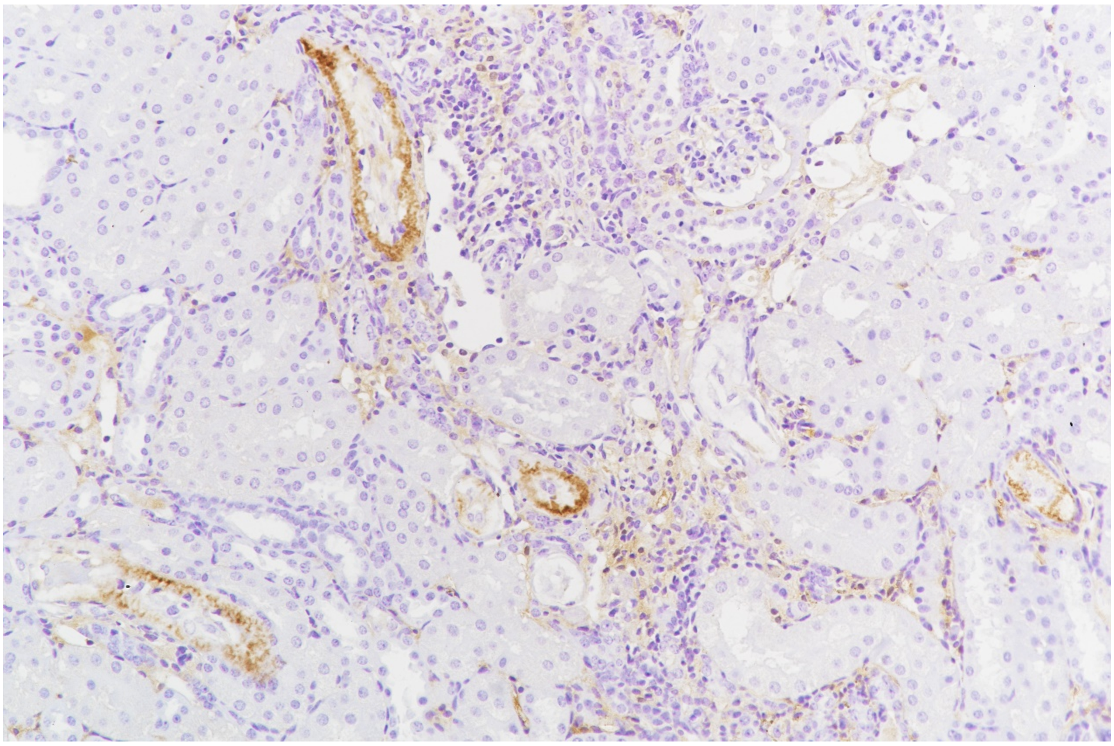

Fig 3B FA for p-STAT1

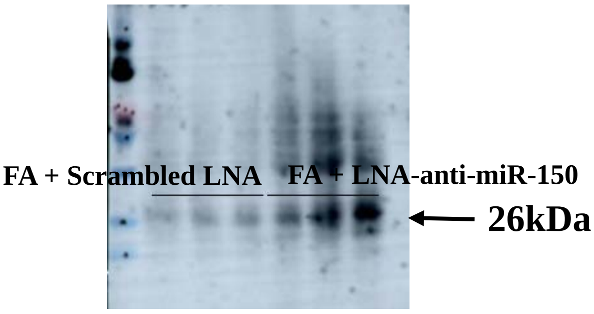

Fig 4A for SOCS1

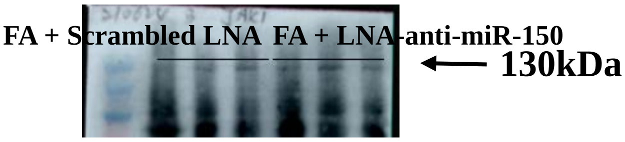

Fig 4A for JAK1

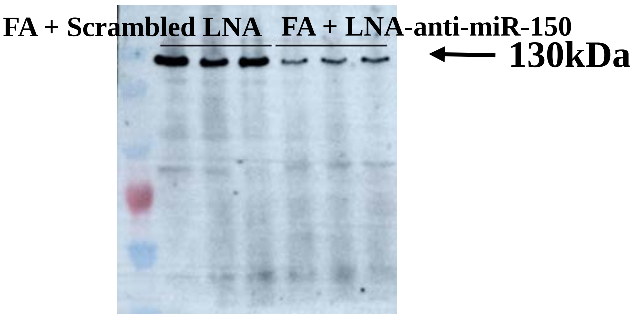

Fig 4A for p-JAK1

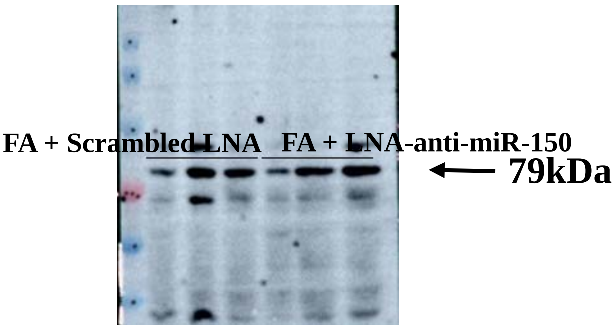

Fig 4A for STAT1

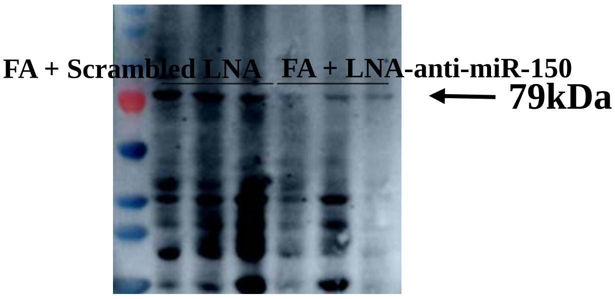

Fig 4A for p-STAT1

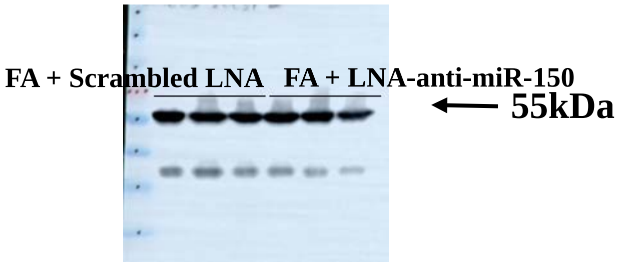

Fig 4A for a-tubulin

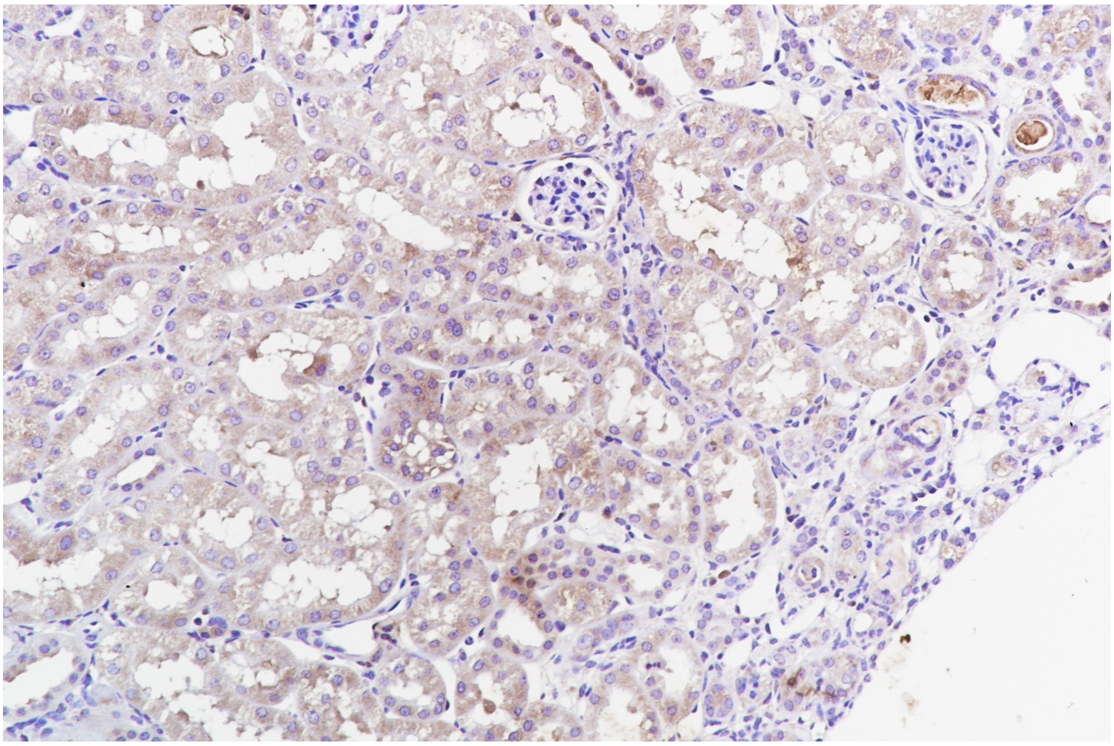

Fig 4B FA + Scrambled LNA for SOCS1

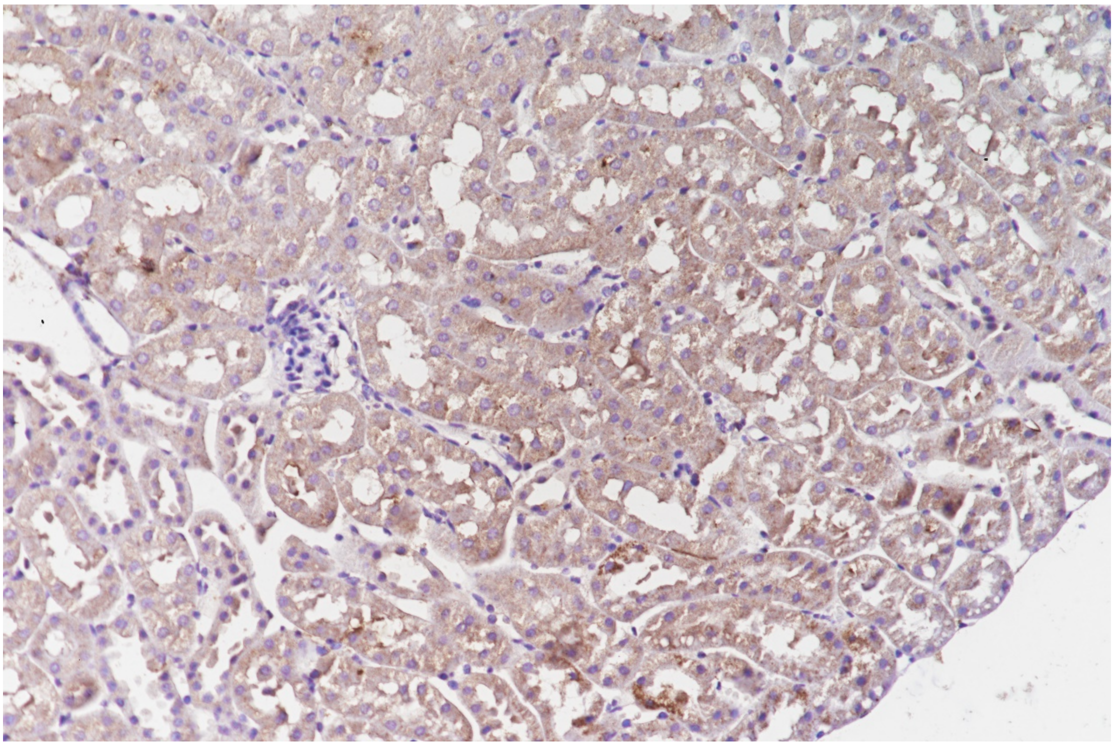

Fig 4B FA + LNA-anti-miR-150 for SOCS1

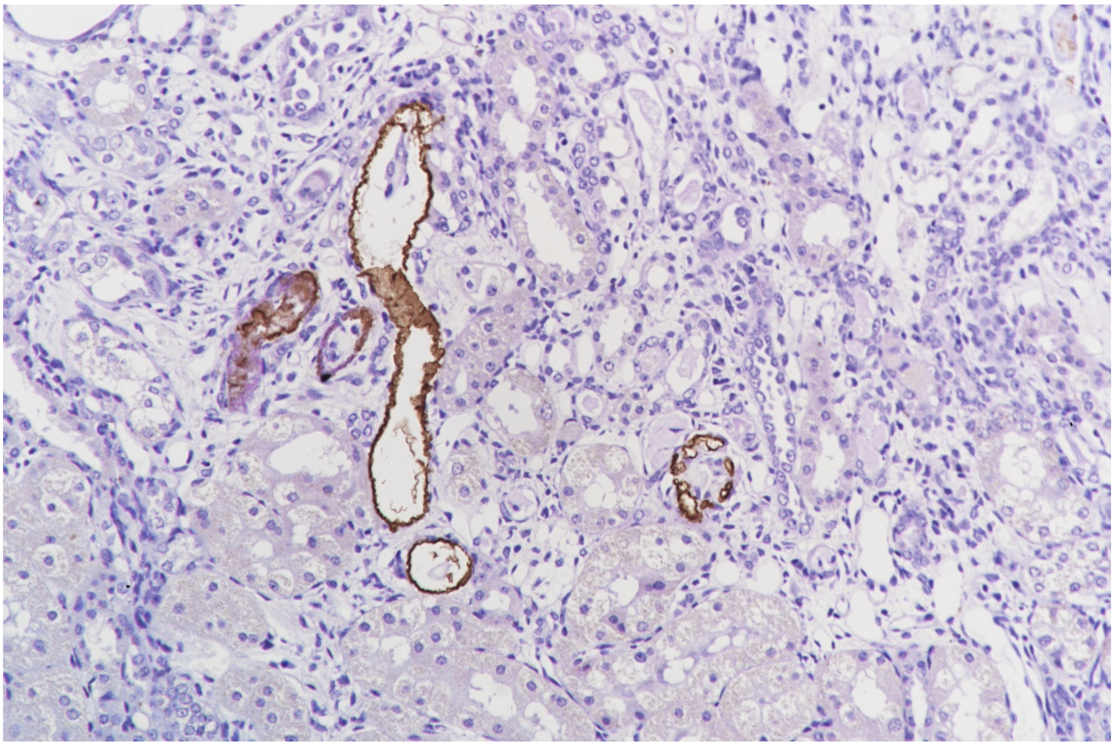

Fig 4B FA + Scrambled LNA for p-JAK1

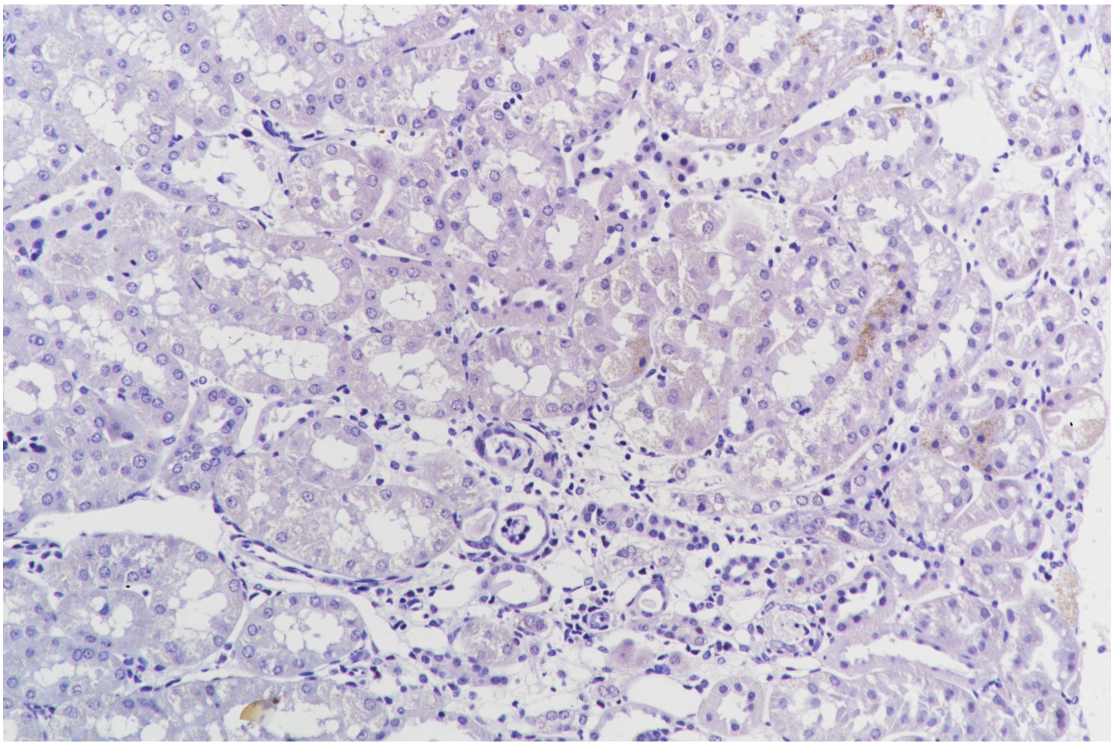

Fig 4B FA + LNA-anti-miR-150 for p-JAK1

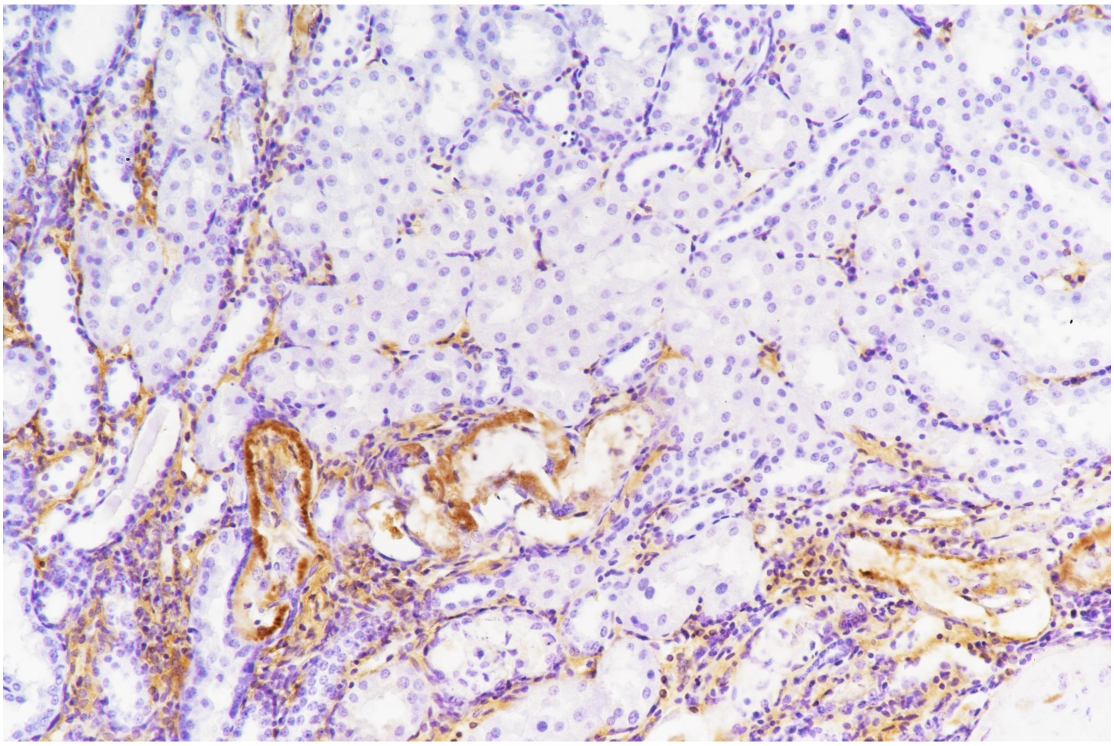

Fig 4B FA + Scrambled LNA for p-STAT1

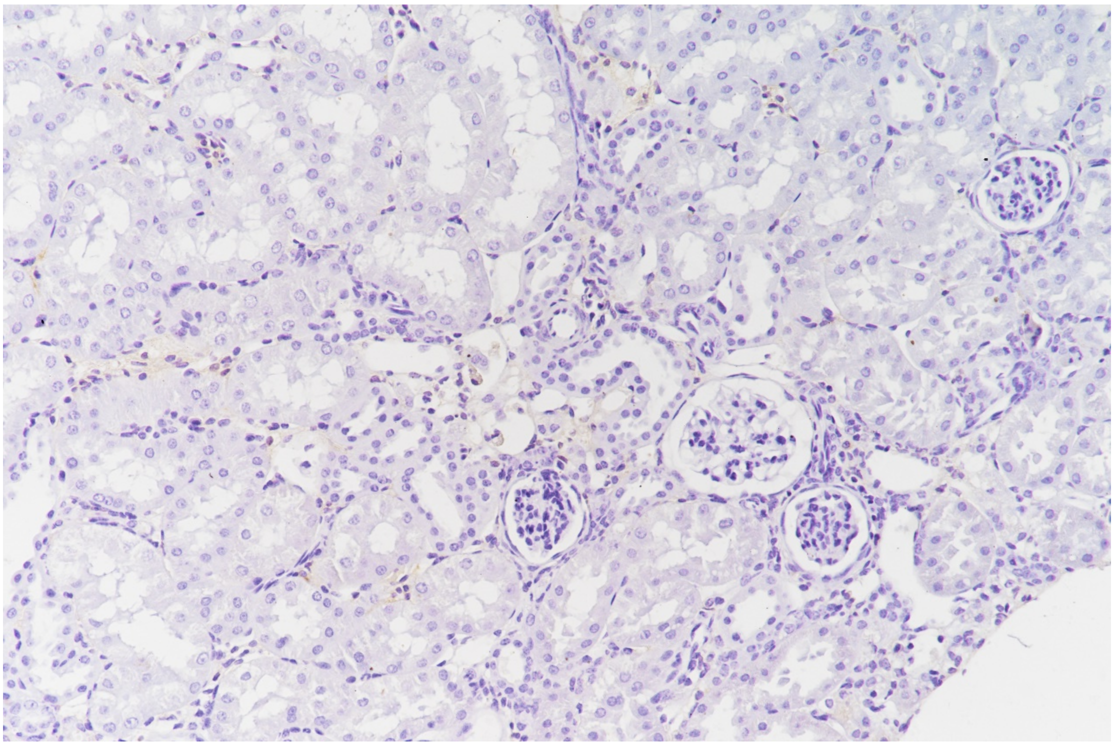

Fig 4B FA + LNA-anti-miR-150 for p-STAT1

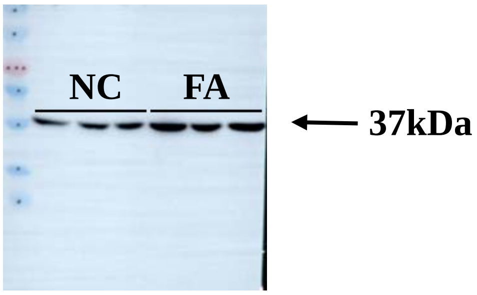

Fig 5A for CD68

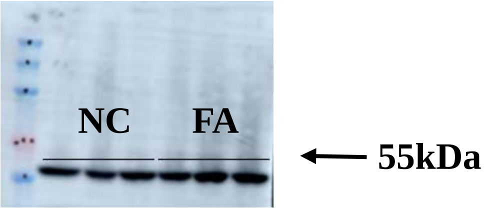

Fig 5A for a-tubulin

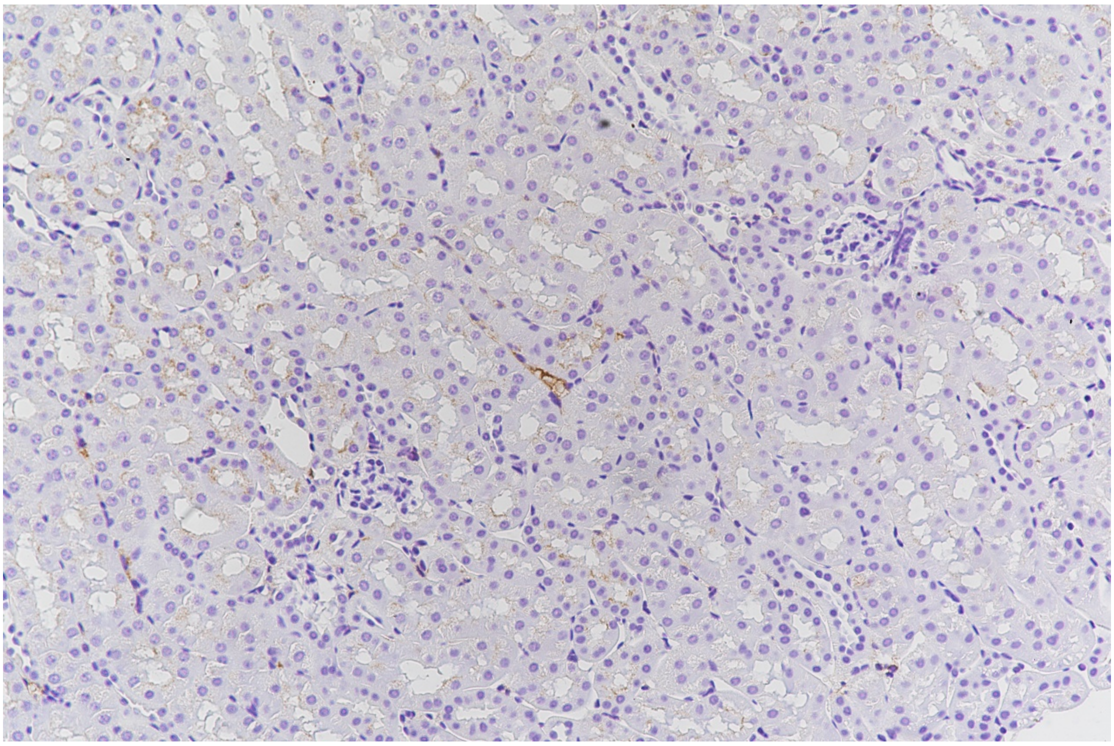

Fig 5B NC for CD68

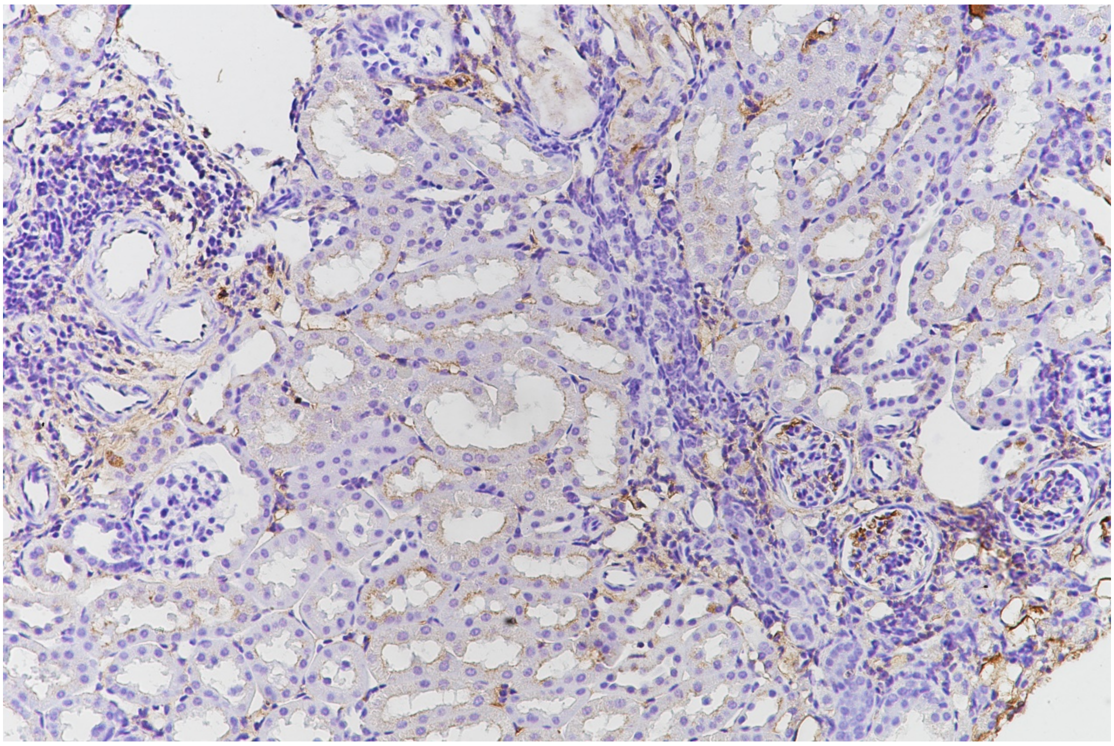

Fig 5B FA for CD68

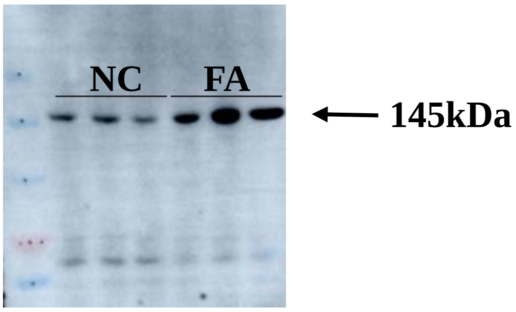

Fig 5C for CD11c

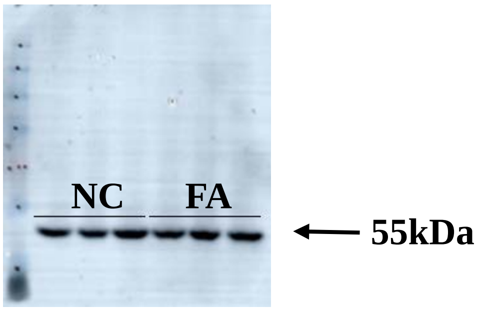

Fig 5C for a-tubulin

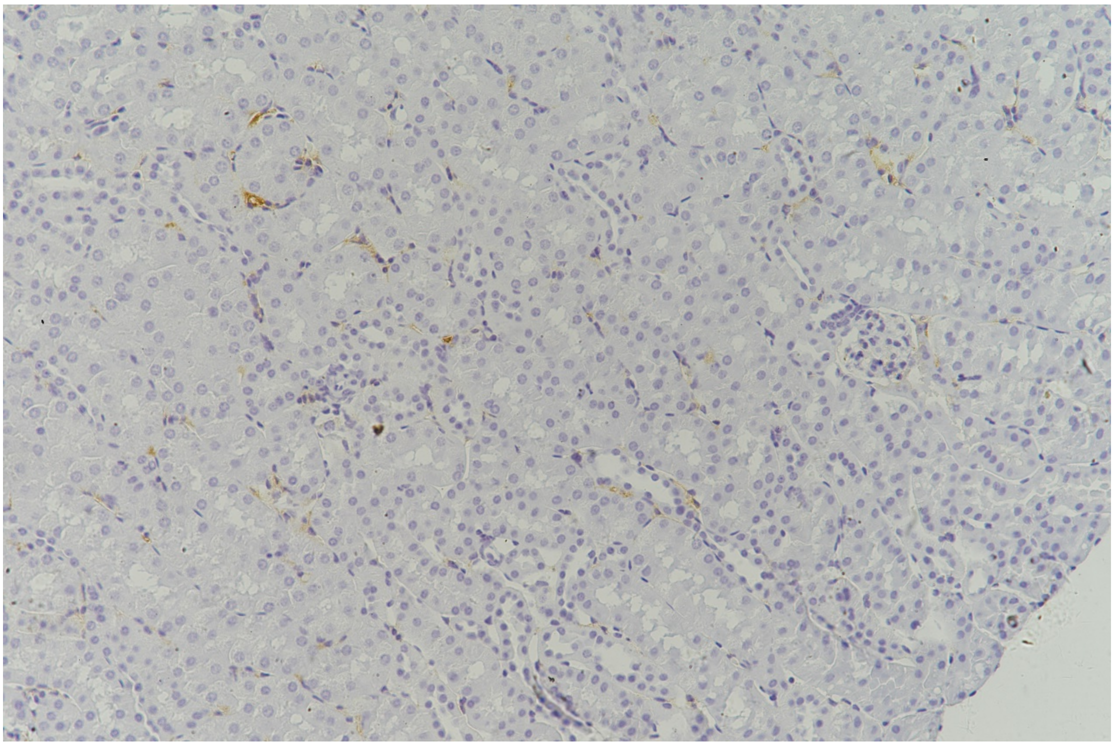

Fig 5D NC for CD11c

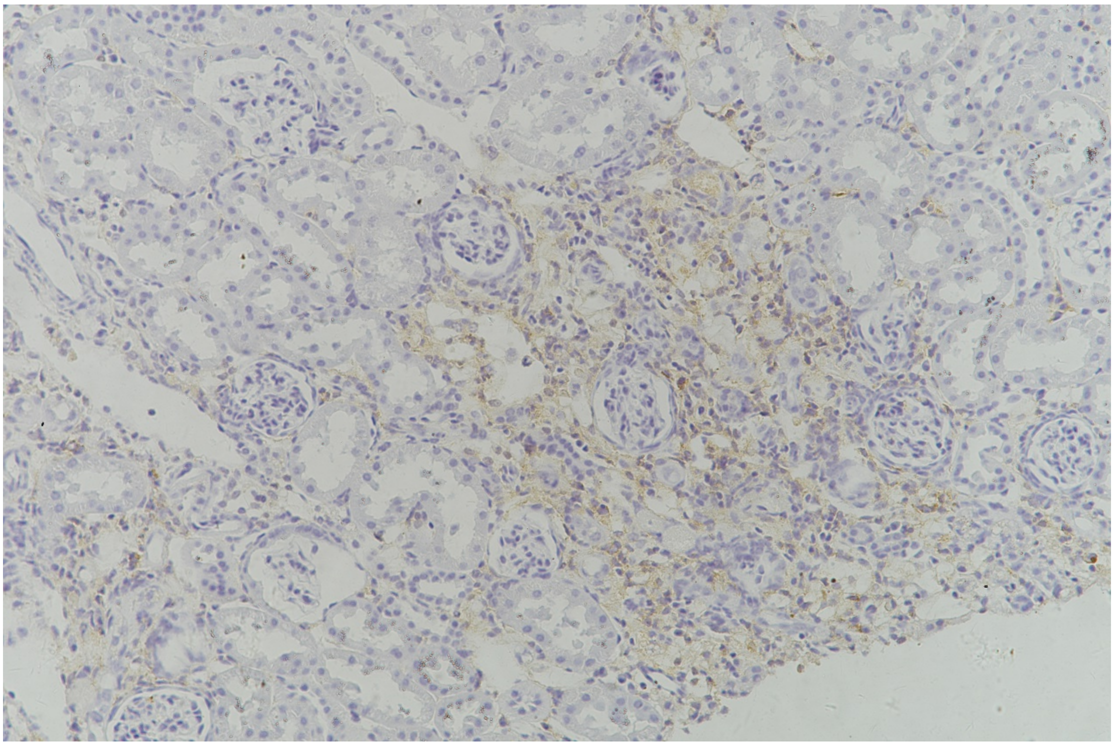

Fig 5D FA for CD11c

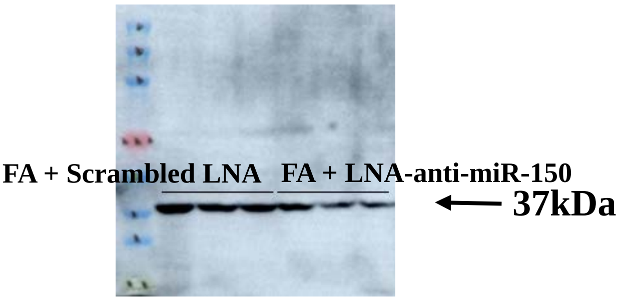

Fig 6A for CD68

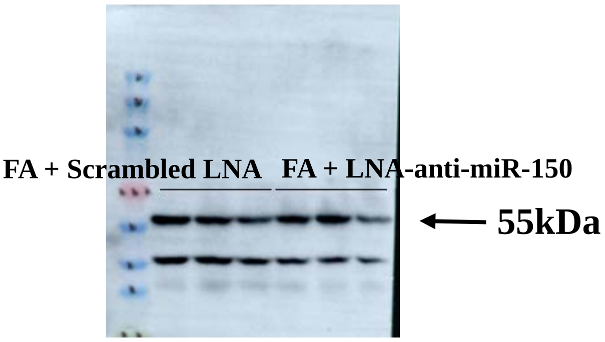

Fig 6A for a-tubulin

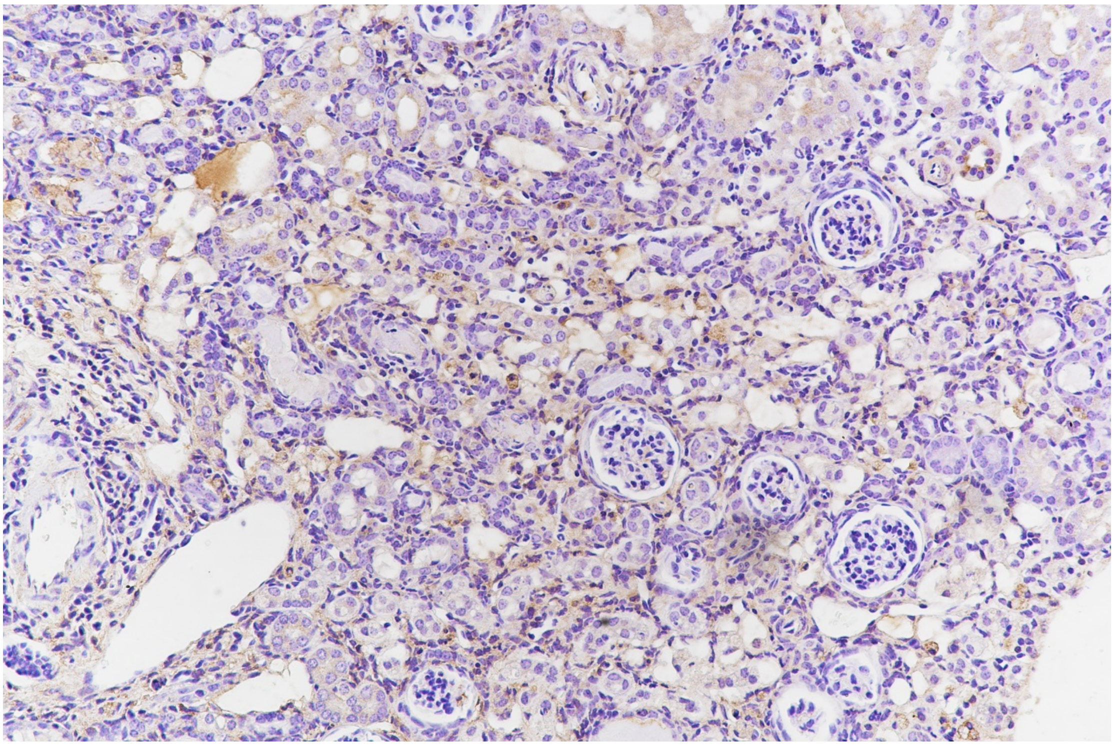

Fig 6B FA + Scrambled LNA for CD68

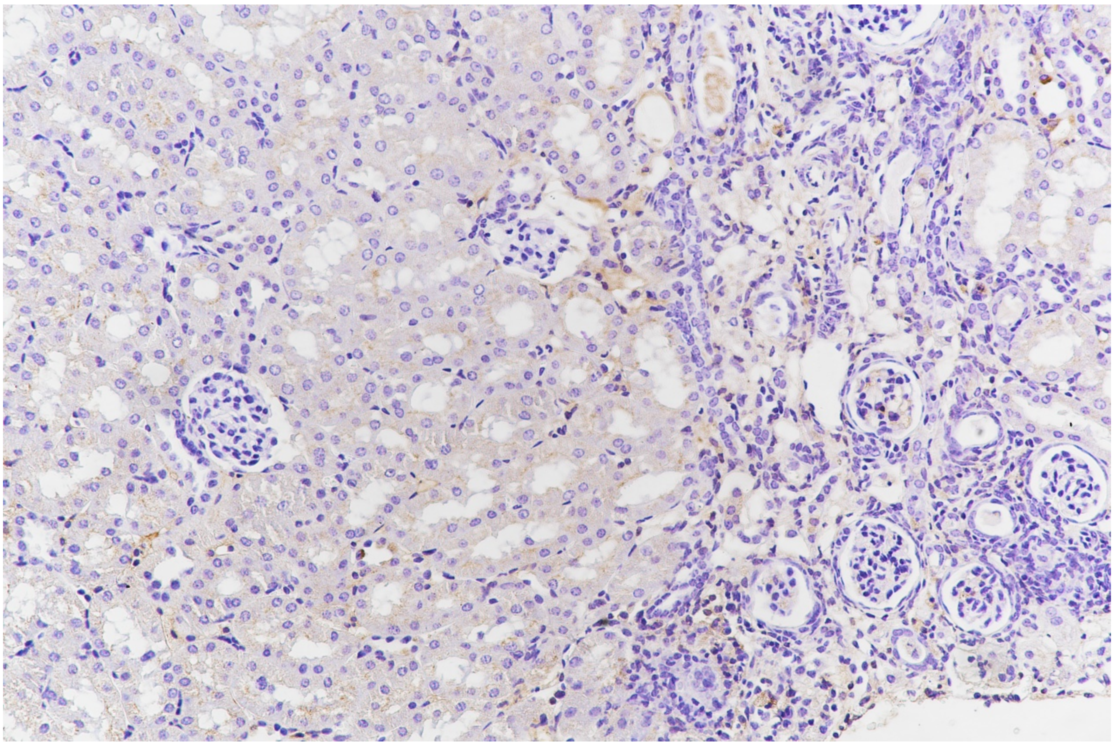

Fig 6B FA + LNA-anti-miR-150 for CD68

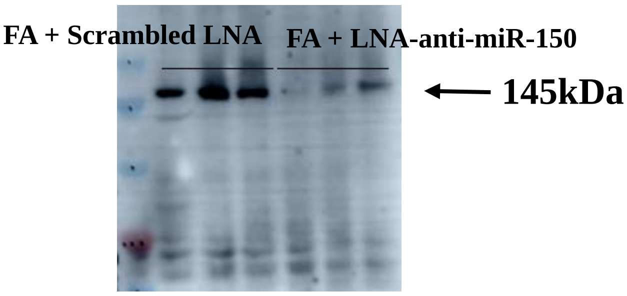

Fig 6C for CD11c

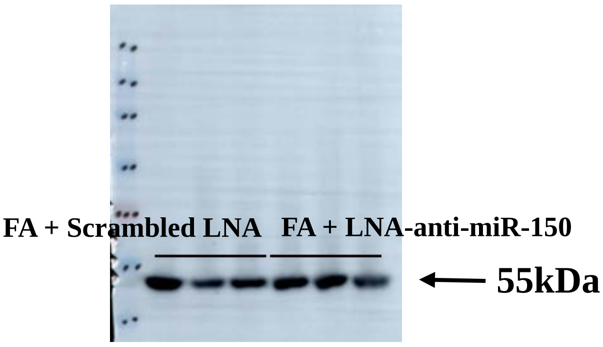

Fig 6C for a-tubulin

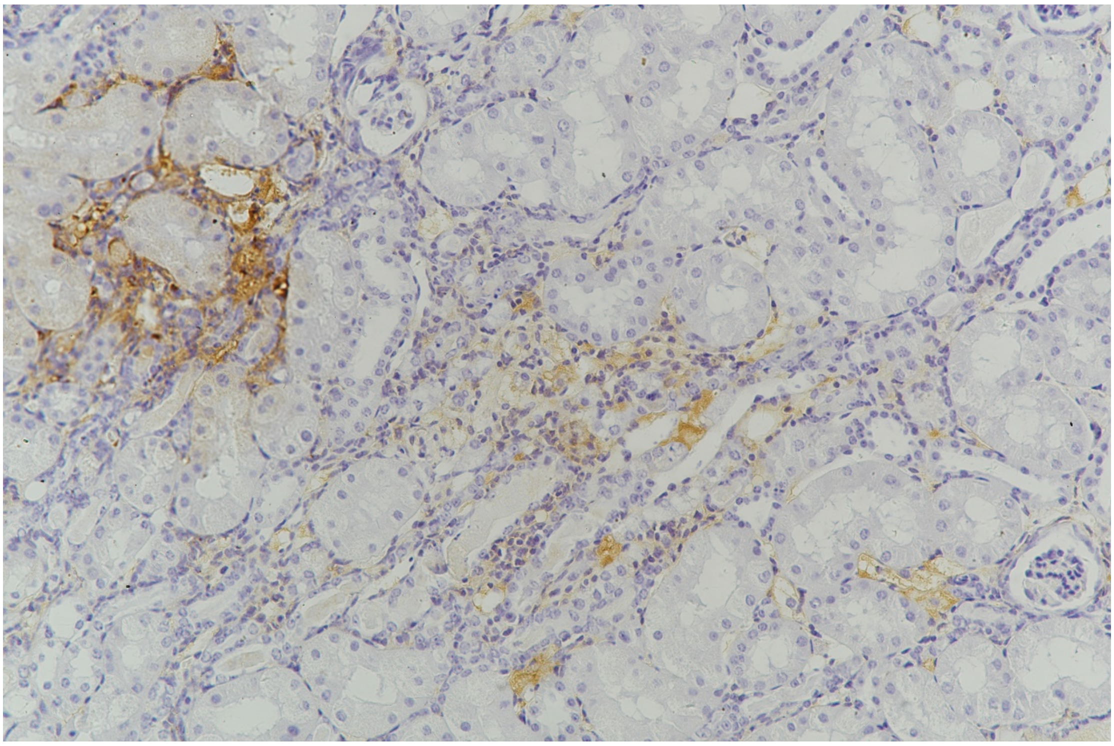

Fig 6D FA + Scrambled LNA for CD11c

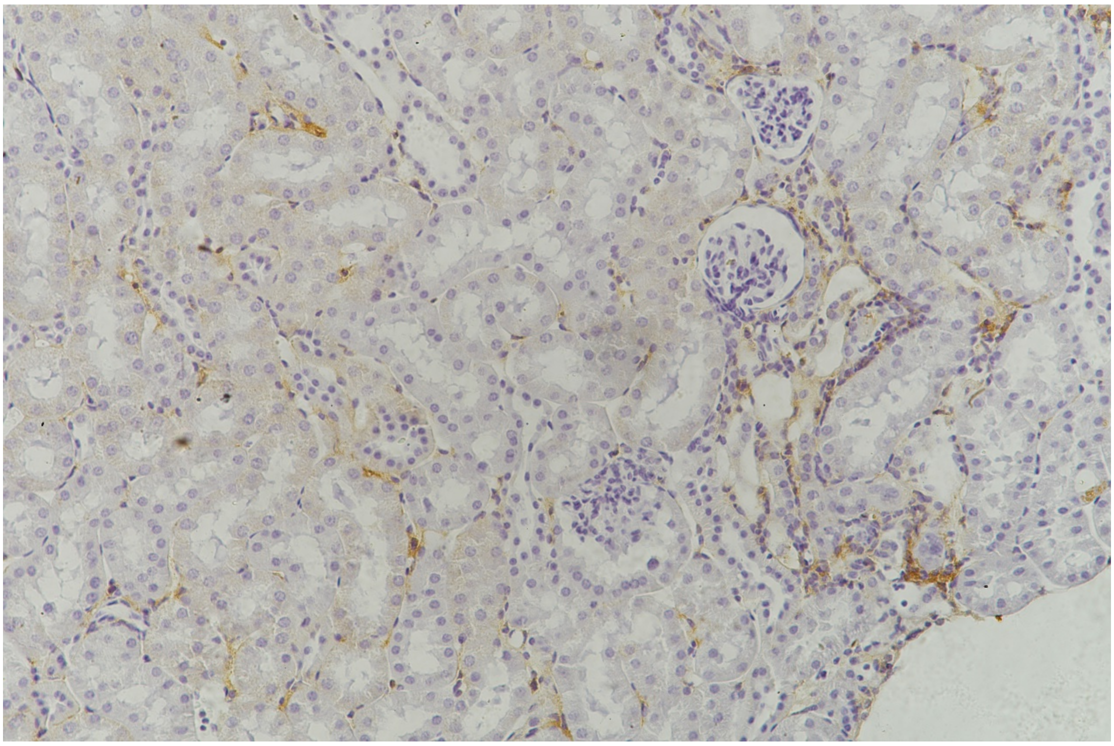

Fig 6D FA + LNA-anti-miR-150 for CD11c
